# Identification and Expression Pattern Analysis of *AsSWEET* Gene Family in *Achnatherum splendens*

**DOI:** 10.3390/ijms26136438

**Published:** 2025-07-04

**Authors:** Ming Hu, Wei Kou, Mingsu Chen, Xiaoying Li, Jingru Wang, Jiahuan Niu, Fei Wang, Hongbin Li, Rong Li

**Affiliations:** 1 Key Laboratory of Oasis Town and Mountain-Basin System Ecology, Xinjiang Production and Construction Corps, Key Laboratory of Xinjiang Phytomedicine Resource Utilization, Ministry of Education, College of Life Sciences, Shihezi University, Shihezi 832003, China; huming@stu.shzu.edu.cn (M.H.); chenmingsu@stu.shzu.edu.cn (M.C.); 13058841181@163.com (X.L.); 18935768685@163.com (J.W.); 17599931126@163.com (J.N.); feiw@shzu.edu.cn (F.W.); 2 College of Life Sciences, Shihezi University, Shihezi 832003, China; weiwei412720@163.com

**Keywords:** sugar transporter, *Achnatherum splendens*, gene family, gene expression, abiotic stress

## Abstract

Sugars Will Eventually Be Exported Transporters (SWEETs) are involved in plant growth and development, particularly in resistance to adverse environments. *Achnatherum splendens* (*Trin.*) *Nevski* exhibits rhizosheath formation and demonstrates notable salt and drought tolerance. We identified 31 sugar transporter family genes (*AsSWEETs*) from the *Achnatherum splendens* genome in the NCBI database and performed bioinformatics analyses, including gene structure, subcellular localization, conserved sequences, promoter cis-acting elements, phylogenetic relationships, and chromosomal localization. The 31 *AsSWEET* genes are distributed across 13 chromosomes, encoding peptides ranging from 375 to 1353 amino acids. Their predicted molecular weights range from 31,499.38 to 109,286.91 Da, with isoelectric points (pI) between 4.78 and 5.21. The aliphatic index values range from 13.59 to 24.19, and the grand average of hydropathicity (GRAVY) values range from 0.663 to 1.664. An analysis of promoter cis-acting elements reveals that all 31 *AsSWEET* genes contain multiple elements related to light, stress, and hormone responses. Subcellular localization predictions indicate that most genes in this family are localized to the plasma membrane or tonoplast, with *AsSWEET12-2* and *AsSWEET3b* localized in chloroplasts and *AsSWEET2b-2* in the nucleus. qRT-PCR results show that *AsSWEET13-1*, *AsSWEET13-3*, and *AsSWEET1a* exhibit upregulated expression in response to salt and drought stress in the roots of *Achnatherum splendens*. These genes may serve as candidate genes for investigating the stress resistance mechanisms of *Achnatherum splendens*. The findings provide a theoretical basis for further research on stress resistance mechanisms and candidate gene identification under salt and drought stress in *Achnatherum splendens*.

## 1. Introduction

Plants encounter various complex and variable environmental challenges throughout their life cycle, including high soil salinity and water scarcity [1], which significantly affect their growth, development, and survival. In plant stress response regulatory networks, Sugars Will Eventually Be Exported Transporters (SWEETs) play vital roles in plant growth, development, and stress responses. They help regulate sugar transport, which is essential for plant survival under adverse conditions. Due to their importance, SWEETs have become a major focus of research in plant stress adaptation [2,3].

Sugars, the primary products of plant photosynthesis [4], serve as both energy sources and regulators of physiological and biochemical processes [4,5,6,7]. Sugar transporter proteins mediate sugar movement between cells and tissues [8,9,10], ensuring appropriate sugar allocation to different organs during growth and under environmental stress [11,12,13]. Changes in sugar transporter expression and activity facilitate plant stress adaptation through three mechanisms: the regulation of cellular water content [11,14,15], enhancement of antioxidant defenses, and activation of stress-related genes via sugar redistribution and metabolic regulation [16].

Sugar transporter proteins are classified into three major groups: sucrose transporters (SUTs), monosaccharide transporters (MSTs), and SWEET (Sugars Will Eventually Be Exported Transporters) proteins [17]. SWEET proteins represent a unique class of transporters that facilitate concentration gradient-dependent sugar diffusion across membranes, a process unaffected by environmental pH [8,18,19]. These transporters regulate intracellular sugar transport, distribution, and storage, processes that are essential for plant growth, development, and stress responses [20]. Under salt stress conditions, certain sugar transporters promote sugar uptake and accumulation in root tissues. This accumulation increases intracellular solute concentrations while decreasing cellular water potential. The resulting osmotic adjustment reduces water efflux from cells, thereby counteracting salt-induced osmotic stress. The accumulated sugars additionally serve as signaling molecules [21], stimulating the expression of stress-responsive genes and initiating defense mechanisms [22]. During drought stress, sugar transporters exhibit two primary functions: the regulation of intracellular sugar concentrations to maintain turgor pressure and support physiological processes, and involvement in stomatal control to reduce water loss and improve water retention capacity.

The SWEET family constitutes a phylogenetically conserved group of sugar transporters characterized by a conserved MtN3/saliva domain architecture. Structural analyses indicate that these proteins typically contain three transmembrane domains [18,23]. Comparative genomic studies reveal distinct domain organizations between eukaryotes and prokaryotes: eukaryotic SWEET proteins contain two MtN3/saliva transmembrane domains, whereas prokaryotic versions possess three transmembrane MtN3/saliva domains. Structural characterization shows that eukaryotic SWEET proteins form seven transmembrane helices through the tandem arrangement of two 3-TMH domains and one TMH (collectively termed the MtN3/saliva domain), generating a characteristic 3–1–3 configuration [24]. Genomic investigations have identified SWEET gene family members in multiple plant species, with 17 members in *Arabidopsis thaliana*, 21 in *Oryza sativa* [23], 52 in *Glycine max* [25], 35 in *Solanum tuberosum* [26], 29 in *Solanum lycopersicum* [27], 28 in *Hylocereus* spp. [24], and 23 in *Sorghum bicolor* [28]. Experimental evidence demonstrates that plant SWEET proteins are involved in various physiological processes, including growth and development [29], glycoconjugate distribution and transport, responses to abiotic and biotic stresses, and regulatory pathways [16,30].

*A. splendens*, a perennial herbaceous species widely distributed in arid and semi-arid regions [31,32,33], possesses a distinctive root system characterized by rhizosheath formation. This specialized morphological adaptation confers exceptional salt and drought tolerance under extreme environmental conditions [34,35], establishing *A. splendens* as a model species for investigating plant stress resistance mechanisms. Under saline conditions, elevated osmotic potential in soil solutions reduces root water uptake efficiency [36]. Concurrently, excessive Na^+^ accumulation induces ionic toxicity [37], compromising cellular homeostasis and impairing essential physiological and metabolic functions [38,39]. Drought stress restricts water availability, causing cellular dehydration and subsequent physiological adaptations, including stomatal closure and photosynthetic suppression [40]. Despite these stressors, *A. splendens* sustains stable growth due to intrinsic stress-adaptive traits. This resilience is indicative of sophisticated regulatory mechanisms governing its stress tolerance.

The rapid development of genomic technologies has enabled the accumulation of extensive biological gene data in the NCBI database, offering valuable resources for plant gene function research. Utilizing this database, we identified 31 sugar transporter genes (*AsSWEETs*) in the *A. splendens* genome. Comprehensive bioinformatics analyses were then conducted, including examinations of gene structure, subcellular localization, conserved domains, promoter cis-acting elements, phylogenetic relationships, and chromosomal distribution, to systematically characterize their potential roles in *A. splendens* growth, development, and stress adaptation. These findings contribute to understanding the molecular basis of stress resistance in *A. splendens* and may facilitate the discovery of stress-tolerant genetic resources with potential applications in crop improvement and ecological restoration.

## 2. Results

### 2.1. Identification and Physicochemical Characterization of the AsSWEET Gene Family

The physicochemical properties of *Achnatherum splendens* (L.) sugar transporter proteins (*AsSWEETs*) were characterized using the ExPASy online tool. As summarized in Table 1, the encoded polypeptides ranged from 375 to 1353 amino acid residues, with molecular masses spanning 31.5 to 109.3 kDa, demonstrating considerable size variation. Theoretical isoelectric point (pI) calculations revealed values between 4.78 and 5.21, which are consistent with acidic proteins. The aliphatic indices (13.59–24.19) and positive GRAVY values (0.663–1.664) confirmed the hydrophobic nature of these transporters.

### 2.2. Secondary Structure Analysis of the AsSWEET Gene Family

**Figure 1:**
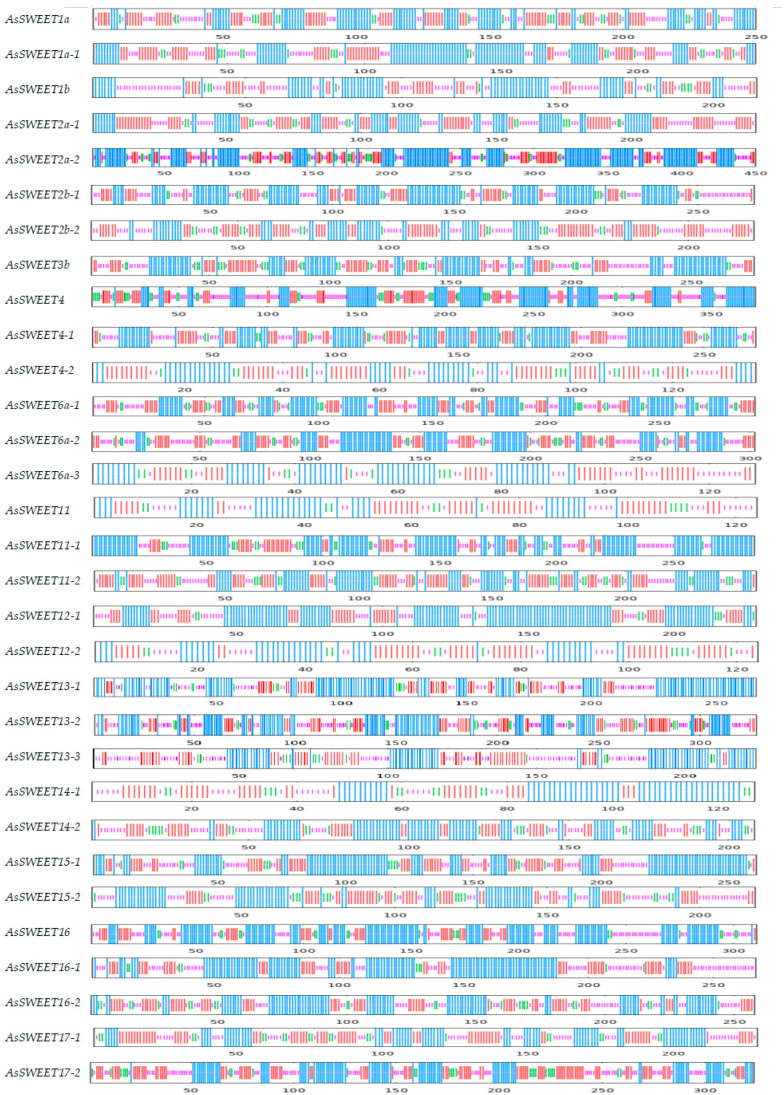
Predicted secondary structure of *AsSWEETs*. The predicted secondary structure of the protein is represented using the following color-coded scheme: blue line, Alpha helix; green line, Beta turn; purple line, random coil; red line, extended strand.

Protein secondary structure denotes the local spatial arrangement of polypeptide backbone atoms, characterized by four principal conformations: α-helices, β-sheets, random coils, and β-turns. SOPMA analysis revealed that α-helices constitute the predominant secondary structural element in *A. splendens AsSWEET* proteins, accounting for 26.23–55.26% of all residues (Table 2). *AsSWEET12-2* displayed the maximum α-helical content (55.26%), while *AsSWEET15-1* showed the minimum (26.23%). Random coils were the second most predominant conformation (20.44–46.68%), with *AsSWEET15-1* containing the highest proportion (46.68%) and AsSWEET2a-2 the lowest (20.44%). Extended strands comprised 15.04–37.98% of the structures. β-sheets were the least abundant structural element, with *AsSWEET12-1* exhibiting minimal representation (0.75%) (Figure 1).

### 2.3. Analysis of Transmembrane Domains in the AsSWEET Family Proteins

**Figure 2:**
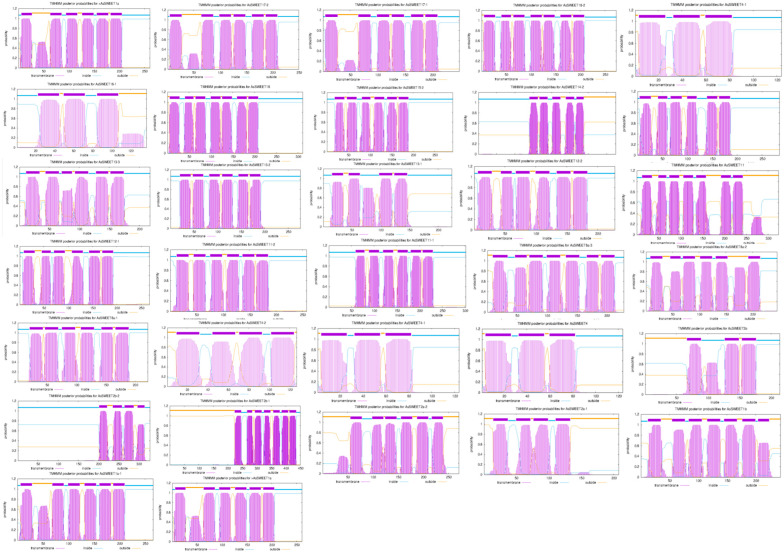
Predicted transmembrane structural domain of AsSWEETs. The predicted transmembrane topology is color-coded as follows: purple for transmembrane domains, blue for intracellular regions, and yellow for extracellular segments. Due to the complexity of presenting all AsSWEET transmembrane domains (TMs) in a single figure, the detailed topological diagrams have been relocated to Supplementary Materials (Supplementary Figure S1) to enable clearer high-resolution analysis.

A transmembrane domain analysis of *A. splendens* AsSWEET proteins was performed using TMHMM 2.0 (Figure 2). The prediction revealed distinct topological architectures: Seven-transmembrane proteins: *AsSWEET16*, *AsSWEET1b*, *AsSWEET2b-1*, *AsSWEET11*, *AsSWEET16-2*, and *AsSWEET6a-3*. *AsSWEET1b* and *AsSWEET11* exhibit an intracellular N-terminus and extracellular C-terminus. The other seven-TM proteins display the opposite orientation (extracellular N-terminus, intracellular C-terminus). Five-transmembrane protein: *AsSWEET14-2* (extracellular N-terminus, intracellular C-terminus). Four-transmembrane proteins: *AsSWEET13-1, AsSWEET2a-1, AsSWEET4-2*, and *AsSWEET2b-2*. *AsSWEET2a-1* shows dual extracellular localization of both termini. Three-transmembrane proteins: *AsSWEET15-1*, *AsSWEET4*, *AsSWEET4-1*, and *AsSWEET3b*. Six-transmembrane proteins: all remaining AsSWEET family members.

### 2.4. Analysis of AsSWEET Gene Structure and Protein Conserved Motifs

**Figure 3:**
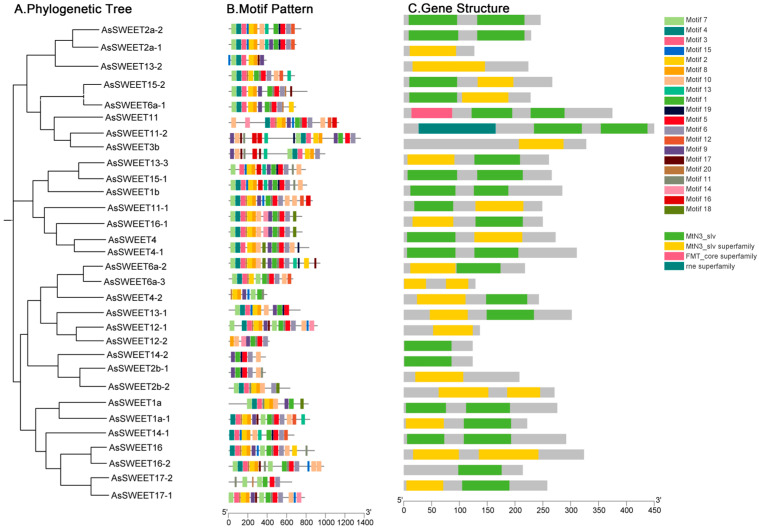
*AsSWEET* family evolutionary tree, motif, and gene structure. (**A**) Phylogenetic tree of *AsSWEET* family members. (**B**) Distribution of conserved motifs in *AsSWEETs* proteins. (**C**) Gene structure of *AsSWEET* family.

To characterize the structural features of *A. splendens AsSWEET* genes, conserved domains, motifs, and phylogenetic relationships were analyzed using NCBI and MEGA7.0, with visualization performed in TBtools (Figure 3). The motif analysis identified 20 distinct motifs with variable distributions across family members. Notably, Motif 7, Motif 2, and Motif 8 were universally conserved among all AsSWEET proteins. Domain architecture analysis (Figure 3C) revealed that most *AsSWEET* proteins contain the characteristic MtN3_slv domain. Two exceptions were observed: AsSWEET11-2 possesses an additional N-terminal MtN3_slv superfamily domain. AsSWEET3b contains an N-terminal FMT_core superfamily domain.

### 2.5. Phylogenetic Analysis of the AsSWEET Gene Family

**Figure 4:**
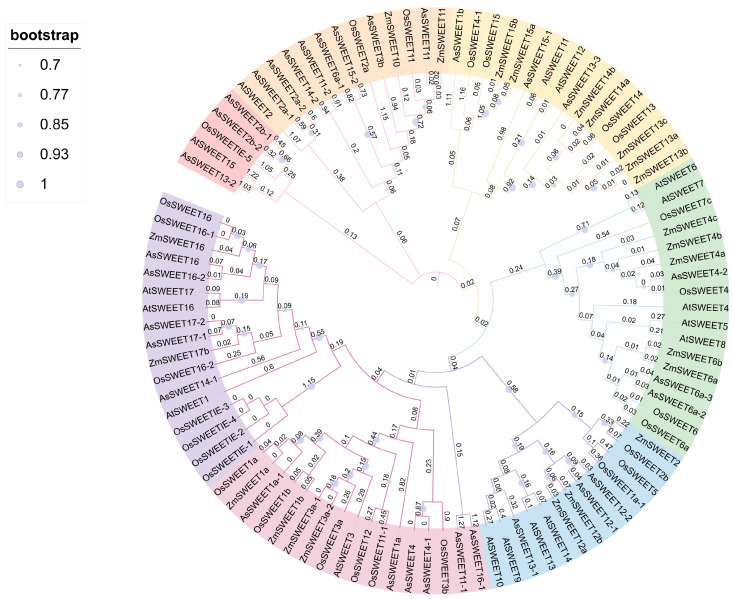
Phylogenetic tree of SWEET gene family members in *Arabidopsis*, *Oryza sativa*, *Zea mays*, and *Achnatherum splendens*.

To elucidate the evolutionary relationships within the *A. splendens* SWEET gene family, we conducted a phylogenetic analysis of protein sequences from 31 *A. splendens* SWEET members, along with their homologs: 17 from *Arabidopsis thaliana (AtSWEET*), 24 from *Oryza sativa* (*OsSWEET*), and 23 from *Zea mays* (*ZmSWEET*), retrieved from the TAIR and NCBI databases. Sequence alignment and phylogenetic tree construction were conducted using MEGA 7.0, with subsequent refinement using iTOL (Interactive Tree Of Life). The phylogenetic analysis revealed distinct evolutionary patterns: the subgroup comprising *AsSWEET2b-2*, *AsSWEET2b-1*, *AsSWEET2a-1*, *AsSWEET2a-2*, *AsSWEET14-2*, *AsSWEET11-2*, *AsSWEET6a-1*, *AsSWEET15-2*, *AsSWEET3b*, *AsSWEET4-1*, *AsSWEET4-2*, *AsSWEET6a-2*, and *AsSWEET6a-3* clustered closely with *Oryza sativa SWEET* family members. *AsSWEET11*, *AsSWEET1b*, *AsSWEET4*, *AsSWEET12-1*, and *AsSWEET12-2* showed stronger affinity to *Zea mays SWEET* proteins. *AsSWEET15-1*, *AsSWEET13-3*, *AsSWEET13-1*, *AsSWEET13-2*, *AsSWEET17-1*, and *AsSWEET17-2* formed a clade with *Arabidopsis SWEET* members. The phylogenetic distribution suggests that the *A. splendens SWEET* gene family comprises multiple subfamilies, with members within each subfamily sharing conserved structural and functional characteristics (Figure 4).

### 2.6. Analysis of Cis-Acting Elements in the Promoter Region of the AsSWEETs Gene Family

**Figure 5:**
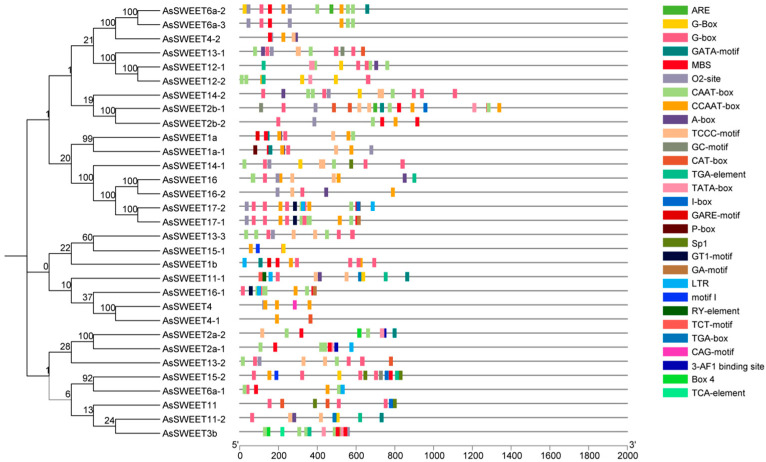
AsSWEET family evolutionary tree and cis-acting elements. Using Plantcare, we identified cis-acting elements in the *AsSWEETs* gene family, and then visualized the results with TBtools. The colored blocks on the right correspond to different regulatory elements, with the scale bar at the bottom indicating sequence length.

To further investigate the transcriptional regulation mechanisms and potential functions of the *A. splendens SWEET* gene family members, the promoter sequences of *AsSWEET* genes were analyzed for cis-acting regulatory elements using the online tools PlantCARE and TBtools (Figure 5). The *AsSWEET* promoter regions contained typical eukaryotic core promoter elements such as TATA-box and CAAT-box, as well as various cis-acting elements associated with abiotic stress responses and transcription factor binding sites. These findings suggest that *AsSWEET* gene expression is regulated by a complex and multifaceted mechanism. Light-responsive elements, including GT1-motif, Box 4, and TCT-motif, indicate that *AsSWEET* expression may be influenced by light. These elements likely interact with transcription factors in light signal transduction pathways to modulate the transcriptional activity of *AsSWEET* genes. Additionally, the presence of abiotic stress-responsive cis-acting elements, such as MBS (drought-inducible element) and ARE (anaerobic response-related regulatory element), implies that *AsSWEET* genes play a crucial role in plant responses to environmental stresses, including drought and hypoxia. These elements may regulate *AsSWEET* expression levels by activating or suppressing transcription in response to stress signals, thereby helping plants adapt to adverse conditions. In summary, the *AsSWEET* promoter regions harbor diverse cis-acting elements and transcription factor binding sites that are modulated by light, phytohormones, and stress signals. This intricate regulatory network allows plants to precisely control *AsSWEET* expression under varying environmental conditions, ensuring optimal growth and developmental adaptability.

### 2.7. Chromosomal Localization Analysis of the AsSWEETs Gene Family

**Figure 6:**
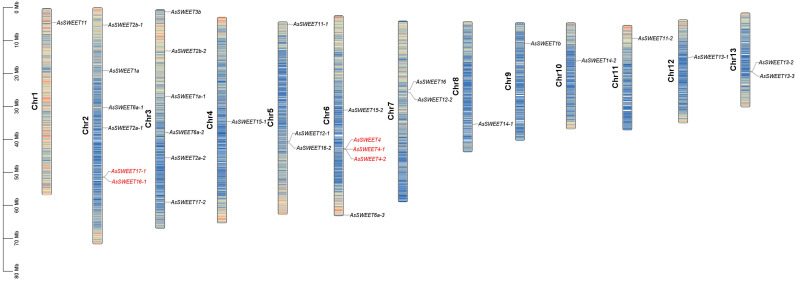
Chromosome localization of *AsSWEET* family. Chromosome names (indicated in black) are placed at the left, with gene names displayed on the right. The scale on the left represents distances in megabases (Mb). The heatmap gradient, ranging from red (high gene density) to blue (low gene density), illustrates the gene distribution on chromosomes, with an estimated inheritance interval of 100 kb. Red font represents same chromosome localization.

A chromosomal localization analysis of *A. splendens SWEET* genes was performed using TBtools to map their physical positions (Figure 6). The distribution pattern revealed three notable gene clusters: Chromosome 2: Co-localization of *AsSWEET17-1* and *AsSWEET16-1*. Chromosome 6: Tandem arrangement of *AsSWEET4*, *AsSWEET4-1*, and *AsSWEET4-2*. Chromosome 13: Shared locus between *AsSWEET13-2* and *AsSWEET13-3*. This clustered genomic distribution pattern suggests that tandem duplication events likely occurred during the evolution of the SWEET gene family.

### 2.8. Subcellular Localization Prediction Analysis of the AsSWEETs Gene Family

Subcellular localization predictions for *AsSWEET* proteins were generated using WOLF PSORT (Table 3). The majority of the family members were predicted to localize to the plasma membrane (58%) or tonoplast (25.8%), consistent with their putative roles in transmembrane sugar transport. Three exceptions were identified: *AsSWEET12-2* and *AsSWEET3b* showed chloroplast localization signatures, and *AsSWEET2b-2* contained nuclear targeting signals.

### 2.9. Expression Analysis of AsSWEETs Gene Family Members Under Abiotic Stress

Analysis of cis-acting elements revealed that in addition to conserved light-responsive elements, *A. splendens SWEET* genes also possess specific elements related to stress responses, such as low-temperature response elements and anaerobic induction elements. Furthermore, considering the abiotic stresses (e.g., temperature, salinity, and drought) faced by *A. splendens* during growth, the seedlings were subjected to salt and drought treatments to investigate the expression patterns of *AsSWEET* family genes under different stresses. The results showed that *AsSWEET* genes exhibited varying expression levels under drought and salt stress.

**Figure 7:**
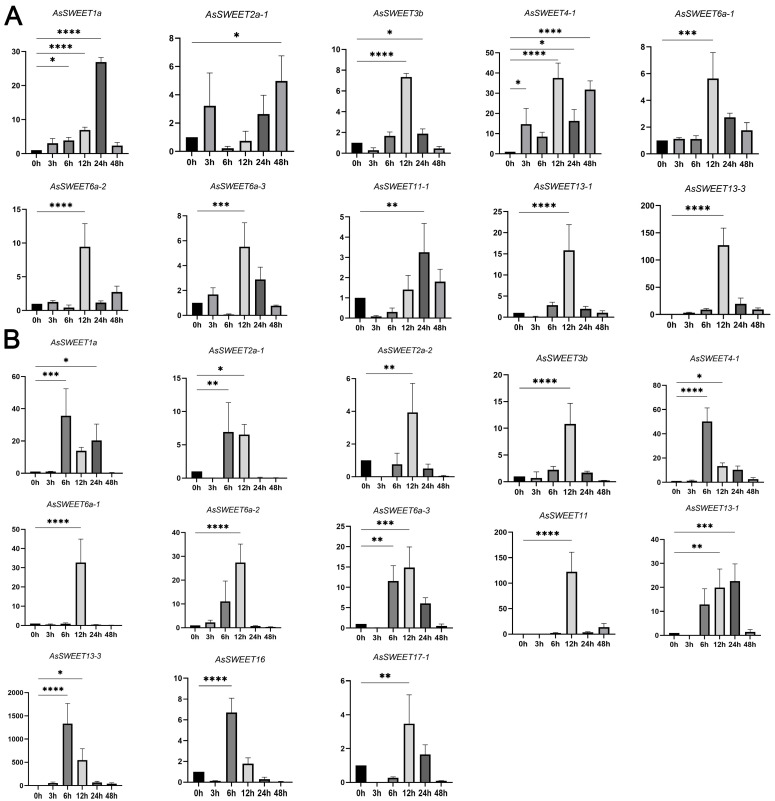
Expression patterns of *AsSWEET* family members under stress. (**A**) Analysis of relative expression levels of *AsSWEET* genes under different drought treatment durations (0/3/6/12/24/48 h) using qRT-PCR. Abscissa is drought treatment time, ordinate is gene-related expression. (**B**) Analysis of relative expression levels of *AsSWEET* genes under different salt treatment durations (0/3/6/12/24/48 h) using qRT-PCR. Abscissa was salt treatment time and ordinate was gene-related expression. Three biological replicates were performed per experiment using GraphPad Prism 9.5 software and visualization of all experimental data. Each bar represents the mean ± SD. The control group was 0 h. Statistics: Significant differences in data were determined with one-way ANOVA and multiple comparison-corrected tests (Dunnett’s test) (relative to controls, * *p* < 0.05, ** *p* < 0.01, *** *p* < 0.001, **** *p* < 0.0001).

In studies of abiotic stress responses, we observed significant expression regulation characteristics in members of the SWEET gene family. Through a qRT-PCR analysis of expression profiles under different stress treatments, the following important findings were obtained: Under simulated drought stress conditions, 10 SWEET transporter genes exhibited distinct temporal expression patterns (Figure 7A). These included *AsSWEET6a-1/2/3, AsSWEET4-1*, and the key sugar transport regulator *AsSWEET11-1*. An expression kinetic analysis revealed that all responsive genes displayed a typical “induction-attenuation” pattern. The responsive genes could be categorized into the following: Early-response group (12 h peak): Seven genes, including *AsSWEET6a-1, AsSWEET6a-2*, and *AsSWEET6a-3*, reached peak expression at 12 h of stress, with a maximum induction of up to 35-fold. Late-response group (24–48 h peak): Three genes (*AsSWEET1a, AsSWEET2a-1*, and *AsSWEET11-1)* showed delayed response characteristics, achieving significant 25-fold expression peaks only during later stress stages (24–48 h).

Under 150 mM NaCl treatment, significant expression changes were detected in 13 SWEET genes (Figure 7B). The expression dynamics could be classified into two typical patterns: Type I (rapid response): Four genes, including *AsSWEET4-1*, reached peak expression at 6 h of stress before gradually declining. Type II (sustained induction): Seven genes, including *AsSWEET16*, showed continuously increasing expression, reaching maxima at 12 h.

Particularly noteworthy was the consistent responsiveness of *AsSWEET1a* and *AsSWEET13-1* under both stress conditions, suggesting that these genes may play core regulatory roles in plant abiotic stress responses. These findings provide important clues for deciphering the molecular mechanisms of the SWEET gene family in plant stress physiology. The differential expression profiles of *AsSWEET* gene family members under drought and salt stress treatments reveal their potential roles in abiotic stress responses in *A. splendens*.

### 2.10. Subcellular Localization

**Figure 8:**
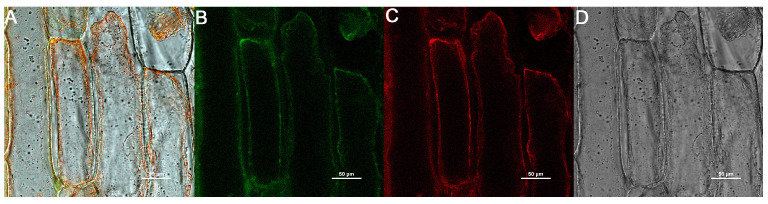
Subcellular localization analysis of *AsSWEET13-3*. Onion epidermal cell expression of *AsSWEET13-3*. Treatment: 0.9% sodium chloride solution. (**A**) Merged field image. (**B**) GFP fluorescence signal. (**C**) Cell membrane marker. (**D**) Bright-field image. The image scale is 50 μm, the excitation wavelength of EGFP is 488 nm, the emission wavelength is 510 nm, the excitation wavelength of the cell membrane maker is 587 nm, and the emission wavelength is 610 nm.

*AsSWEET13-3* was selected for subcellular localization analysis based on its distinctive stress-responsive expression pattern. Quantitative RT-PCR revealed that this gene exhibited the most significant differential expression under both drought and salt stress conditions (Figure 8), suggesting its potential functional importance in abiotic stress responses. To validate the subcellular localization of *AsSWEET13-3*, co-transfection experiments were performed in onion epidermal cells using a plasma membrane marker. Confocal microscopy confirmed the plasma membrane localization of *AsSWEET13-3*, which was aligned with bioinformatic predictions. This localization pattern is consistent with its biological function as a sugar transporter and provides an important cytological basis for subsequent studies on its transport function.

## 3. Discussion

The *SWEET* protein family is evolutionarily conserved across plant species and regulates diverse physiological processes. Genomic studies have identified and characterized *SWEET* members in multiple species, including *Arabidopsis thaliana*, *Oryza sativa*, *Triticum aestivum*, *Avena sativa* [41], *Capsicum annuum* [42], *Glycine max*, *Camellia oleifera* [43], *Lycium barbarum* [44], *Vitis vinifera* [45], *Triticosecale* [46], and *Zea mays* [47]. Functional analyses demonstrate that *SWEET* genes participate in essential biological processes, including vegetative growth, endosperm development, and abiotic stress responses.

A genomic analysis identified 31 *SWEET* genes in *Achnatherum splendens*. Structural characterization revealed that *AsSWEET* proteins are primarily composed of α-helices (26.23–55.26%), with lower proportions of extended strands (15.04–37.98%), β-turns, and random coils (20.44–46.68%). Chromosomal distribution analysis demonstrated dispersion across all 13 chromosomes, with no evidence of tandem duplication, consistent with the evolutionary conservation observed in other plant *SWEET* families. Physicochemical analysis showed that *AsSWEET* proteins range from 375 to 1353 amino acid residues, exhibit a grand average of hydropathicity (GRAVY) values of 0.663–1.664, and possess isoelectric points (pI) between 4.78 and 5.21. These characteristics confirm their classification as hydrophobic, acidic proteins. Subcellular localization predictions indicated predominant plasma membrane targeting (58%), with exceptions showing chloroplast (*AsSWEET12-2*, *AsSWEET3b*) and nuclear (*AsSWEET2b-2*) localization patterns.

Promoter cis-acting elements play pivotal roles in transcriptional regulation and functional diversification. An analysis of *A. splendens SWEET* gene promoters revealed distinct cis-regulatory elements that can be categorized into three functional groups: stress-responsive elements, drought-inducible (MBS); hormone-responsive elements, Gibberellin (P-box), Auxin (TGA-element), and Salicylic acid (TCA-element); And growth-related elements, light-responsive (G-box, Box 4). This regulatory architecture suggests that *SWEET* genes participate in stress adaptation, phytohormone signaling, and developmental processes. Experimental validation by qRT-PCR demonstrated that 13/31 *AsSWEET* genes exhibit significant transcriptional responses (*p* < 0.05) to salt (150 mM NaCl) and drought (20% PEG-6000) stresses, supporting their functional involvement in A. splendens stress tolerance mechanisms. These genes are highly likely to be key regulatory factors in the stress resistance mechanisms of *A. splendens*. Under salt stress conditions, they may enhance sugar uptake and transport in roots through up-regulated expression, leading to the increased accumulation of soluble sugars within cells. This process improves cellular osmoregulation capacity, reduces water loss, and maintains turgor pressure and normal physiological functions. During drought stress, these genes may participate in regulating sugar signaling pathways in roots, activating downstream stress-responsive gene expression to initiate plant defense responses, such as promoting root growth and enhancing membrane stability. However, the specific mechanisms by which these genes perceive stress signals and regulate sugar transport and downstream gene expression through signal transduction pathways require further in-depth investigation.

While this study has yielded valuable findings, several limitations should be acknowledged. First, the current research primarily relies on bioinformatics analysis and gene expression profiling, lacking direct functional validation of AsSWEET genes at the molecular, cellular, and physiological levels. Future studies could employ gene editing technologies (e.g., CRISPR-Cas9) to generate *A. splendens* knockout or overexpression lines for elucidating the precise roles of these genes in stress resistance mechanisms. Second, the interactions between *AsSWEET* genes and other stress-related genes/metabolic pathways, as well as their dynamic regulation patterns under varying environmental conditions (e.g., different salt concentrations, drought intensities and durations), remain unexplored. Subsequent research could integrate multi-omics approaches (proteomics, metabolomics, etc.) to comprehensively decipher the molecular regulatory networks underlying A. splendens’ stress tolerance, providing a more solid theoretical foundation for exploiting plant stress-resistant gene resources and improving crop resilience.

In conclusion, our systematic analysis of the *AsSWEET* gene family in *A. splendens* provides important insights into plant stress adaptation mechanisms and identifies promising research directions. Further functional characterization of these genes and their regulatory mechanisms may offer novel strategies and genetic resources for agricultural production and ecological restoration. Future research directions should include the functional validation through transgenic approaches, detailed characterization of substrate specificities, and investigation of post-translational regulation mechanisms under stress conditions.

## 4. Materials and Methods

### 4.1. Plant Materials

The seeds of *A. splendens* used in this study were collected from the Xinjiang Uygur Autonomous Region. After collection, the seeds were dried in an oven at 37 °C, and plump grains were selected. The glumes were removed, and the seeds were sequentially sterilized by rinsing three to five times with sterile water, followed by three washes with 75% ethanol (90 s each), another three to five rinses with sterile water, and a 6 min treatment with 1% sodium hypochlorite. Finally, the seeds were washed five to six times with sterile water and placed on ½ MS solid medium for germination and root development. After 60 days of germination, the seedlings were subjected to experimental treatments and sampling.

### 4.2. Experimental Methods

#### 4.2.1. Identification and Physicochemical Characterization of the SWEET Gene Family in *A. splendens*

In this study, the *SWEET* protein sequences of *Arabidopsis thaliana*, *Zea mays*, and *Oryza sativa* were downloaded from the NCBI online database (https://www.ncbi.nlm.nih.gov/ (accessed on 1 May 2024)). The Hidden Markov Model (HMM) profiles of *SWEETs* (PF03083, IPR047664, IPR004316) were obtained from the Pfam database (http://pfam-legacy.xfam.org/ (accessed on 1 May 2024)). Using this model and the TBtools software (version 2.89.0.0), a genome-wide search was performed on the protein sequences of *A. splendens*, resulting in the identification of 31 candidate *SWEET* genes. These 31 candidate genes were further validated for conserved domains using the Pfam online tool and NCBI-CDD (https://www.ncbi.nlm.nih.gov/Structure/bwrpsb/bwrpsb.cgi (accessed on 1 May 2024)) (parameters: E-value threshold, 0.01; maximum number of hits, 500), ensuring that only those containing the characteristic SWEET domains were retained. Subsequently, the physicochemical properties of the identified A. splendens SWEET gene family members were analyzed using the ExPASy online tool (https://web.expasy.org/protparam/ (accessed on 1 May 2024)).

#### 4.2.2. Secondary Structure Analysis of the SWEET Gene Family in *A. splendens*

The secondary structures and their compositional percentages of the 31 identified *SWEET* gene family members in *A. splendens* were analyzed using the SOPMA (Self-Optimized Prediction Method with Alignment) online tool (http://pbil.ibcp.fr/ (accessed on 1 May 2024)). This analysis provided predictions of different secondary structural elements, including α-helices, β-sheets, turns, and random coils, for each *SWEET* protein sequence.

#### 4.2.3. Transmembrane Domain Analysis of *A. splendens* SWEET Proteins

The transmembrane domains of *AsSWEET* proteins were predicted using the TMHMM 2.0 online tool (https://services.healthtech.dtu.dk/services/TMHMM-2.0/ (accessed on 1 June 2024)). This analysis helped identify the number and topology of transmembrane helices, which are characteristic features of sugar transporter proteins.

#### 4.2.4. Gene Structure and Protein Motif Analysis of *A. splendens* SWEET Family

Conserved protein motifs were analyzed using the MEME suite (https://meme-suite.org/index.html (accessed on 1 June 2024)), while gene structures (exon–intron organization) were examined using NCBI-CDD (https://www.ncbi.nlm.nih.gov/Structure/bwrpsb/bwrpsb.cgi (accessed on 1 June 2024)). The results were visualized using TBtools software to compare structural patterns among different *AsSWEET* members.

#### 4.2.5. Phylogenetic Analysis of *A. splendens* SWEET Gene Family

A phylogenetic tree was constructed using MEGA11.0 software, incorporating *SWEET* protein sequences from *A. splendens*, *Arabidopsis thaliana*, and *Oryza sativa*. The Neighbor-Joining (NJ) method with 1000 bootstrap replicates was employed to establish evolutionary relationships. The resulting tree was further annotated and visualized using the iTOL online platform (https://itol.embl.de/ (accessed on 1 June 2024)).

#### 4.2.6. Promoter cis-Acting Element Analysis of *A. splendens* SWEET Genes

The 2000 bp upstream sequences from the translation start site of *AsSWEET* genes were extracted using TBtools. Putative cis-regulatory elements were predicted using PlantCARE (http://bioinformatics.psb.ugent.be/ (accessed on 1 June 2024)), followed by visualization of stress- and hormone-related elements through TBtools.

#### 4.2.7. Chromosomal Localization of *A. splendens* SWEET Genes

Genomic positions of *AsSWEET* genes were mapped onto chromosomes based on the genome annotation file. The chromosomal distribution was visualized using TBtools.

#### 4.2.8. Subcellular Localization Prediction of *A. splendens* SWEET Gene Family Members

The subcellular localization of *AsSWEET* proteins was predicted using WoLF PSORT (https://www.genscript.com/wolf-psort.html (accessed on 1 June 2024)), a sophisticated online tool that analyzes protein amino acid sequences to predict their probable cellular compartments. 

#### 4.2.9. Abiotic Stress Expression Analysis of SWEET Gene Family Members in *A. splendens*

In this experiment, we treated *A. splendens* with 500 mmol/L D-mannitol and 100 mmol/L NaCl to simulate the drought and salt stresses, respectively. The roots of plants treated at 0, 3, 6, 12, 24, and 48 h were taken to be treated with liquid nitrogen quick freezing. RNA was extracted using the Polysaccharides and Polyphenols Plant Total RNA Extraction Kit from TIANGEN(TIANGEN Biotech (Beijing) Co., Ltd., Beijing, China), and the RNA integrity was examined by 1.2% agarose gel electrophoresis. The concentration of RNA was determined and stored in the refrigerator at −80 °C. The RNA was reverse-transcribed into cDNA, diluted 10-fold as a template, and then analyzed using fluorescence quantitative PCR. The quantitative fluorescence reaction system and program were set up according to the instruction manual of the TIANGEN kit, and the internal reference gene was As1299. Three technical replicates were set up for each sample, and the relative expression was calculated by 2^−ΔΔCt^ [48]. The relevant primers are shown in Supplementary Materials Table S1.

#### 4.2.10. Construction of Plant Expression Vector

Under both drought and salt stress conditions, AsSWEET13-3 exhibited the most pronounced induction, strongly suggesting its functional specialization in plant abiotic stress tolerance. Therefore, AsSWEET13-3 was selected for subcellular localization analysis. Homologous arm primers were designed using the Vazyme online tool (https://crm.vazyme.com/cetool/singlefragment.html (accessed on 1 June 2024)) to construct the *AsSWEET13-3*:pCAMBIA1300 vector. The assembled plant overexpression vector was then introduced into onion cells via Agrobacterium-mediated vacuum infiltration for subcellular localization analysis. The pCAMBIA1300 vector was double-digested with *Kpn I* and *Xba I*, followed by gel purification. The full-length target gene was cloned and ligated to the vector using homologous recombination. The construct was transformed into DH5α competent cells and plated on LB solid medium containing kanamycin. After 48 h, positive single colonies were selected for verification and sent to Sangon Biotech (Shanghai, China) for sequencing (the detailed procedures are provided in Supplementary Materials Tables S2–S4). Sequence alignment analysis was performed using SnapGene (version 6.0.2.0). Correctly aligned colonies were amplified, and plasmids were extracted using a plasmid extraction kit according to the manufacturer’s instructions to obtain the *AsSWEET13-3* positive plasmid. The validated 35S:*AsSWEET13-3*-GFP recombinant plasmid was transformed into Agrobacterium GV3101 via the freeze–thaw method and plated on LB solid medium containing triple antibiotics (Gen 50 μg·mL^−1^, Kan 50 μg·mL^−1^, Rif 50 μg·mL^−1^). After incubation at 28 °C for 48 h, single colonies were selected for PCR verification. The confirmed bacterial cultures were preserved for subsequent use.

#### 4.2.11. Subcellular Localization

Fresh onions were purchased, and their roots were soaked in water. The recombinant plasmid-carrying Agrobacterium tumefaciens was activated in liquid LB medium containing gentamicin, rifampicin, and kanamycin sulfate, then incubated in a shaker at 100× *g* and 28 °C for 12 h. The bacterial cells were collected by centrifugation at 5000 rpm for 10 min. Pre-cultured onion bulbs were cut into 1 cm^3^ pieces and placed in MS liquid medium containing the resuspended bacterial solution (100 mmol·L^−1^ MES, 10 mmol·L^−1^ MgCl_2_, 100 μmol·L^−1^ AS). The bacterial suspension was adjusted to an OD_600_ of 1.0 and incubated on a shaker at 100× *g* and 28 °C for 30 min. After incubation, the onion pieces were blotted dry with filter paper, placed face-up on MS solid medium lined with filter paper, and cultured in the dark for 48 h. Before observation, the onion pieces were rinsed with ddH_2_O to remove residual Agrobacterium on the surface. Plasmolysis was induced using a 0.9% sodium chloride solution, and the samples were observed and imaged under a confocal laser scanning microscope.

## 5. Conclusions

To adapt to long-term environmental pressures, plants undergo genetic changes that enhance their survival and reproductive success. These adaptive responses are crucial for plant resilience and represent a core aspect of stress tolerance evolution. In this study, we identified 31 *AsSWEET* genes in the *A. splendens* genome using bioinformatics approaches. Most of these genes are localized to the plasma membrane or vacuolar membrane and possess the characteristic MtN3_slv domain. The *AsSWEET* family proteins primarily consist of secondary structures such as α-helices, β-sheets, and random coils.

The 31 *AsSWEET* genes are distributed across 13 chromosomes, and their promoters contain numerous cis-acting elements associated with light responsiveness, stress adaptation, and hormone signaling, including MBS and ARE motifs. Notably, *AsSWEET13-1*, *AsSWEET13-3*, and *AsSWEET1a* exhibited strong responses to salt and drought stress in the roots of A. splendens. These findings demonstrate that the *AsSWEET* gene family plays a critical role in the stress resistance mechanisms of *A. splendens*, laying a foundation for further research into how this species adapts to saline and arid environments.

## Figures and Tables

**Table 1:** Physicochemical properties of *AsSWEETs*.

Gene Name	Protein/ (aa)	MW(Da)	PI	Instability Index	Aliphatic Index	GRAVY
*AsSWEET1a*	785	65,294.44	5.05	50.48	17.43	0.736
*AsSWEET1a-1*	801	66,709.30	5.04	54.29	16.73	0.728
*AsSWEET1b*	732	61,692.02	5.08	48.68	15.85	0.829
*AsSWEET2a-1*	627	52,648.77	5.11	48.12	20.26	0.963
*AsSWEET2a-2*	816	69,883.04	5.07	43.95	18.38	0.915
*AsSWEET2b-1*	1353	109,286.91	4.99	41.24	18.99	0.839
*AsSWEET2b-2*	987	79,015.14	5.06	41.72	20.67	0.980
*AsSWEET3b*	645	53,983.53	5.14	35.81	24.19	0.949
*AsSWEET4*	375	31,499.38	5.18	57.86	21.07	0.933
*AsSWEET4-1*	375	31,521.36	5.18	56.29	18.93	0.939
*AsSWEET4-2*	384	33,170.84	5.14	59.34	14.06	0.949
*AsSWEET6a-1*	669	56,383.84	5.08	54.43	19.73	0.817
*AsSWEET6a-2*	741	63,173.95	5.05	53.99	18.35	0.786
*AsSWEET6a-3*	690	58,671.54	5.07	53.71	19.28	0.79
*AsSWEET11*	975	83,415.84	4.98	52.94	16.10	0.849
*AsSWEET11-1*	909	77,606.83	5.00	55.62	16.28	0.837
*AsSWEET11-2*	777	66,672.56	5.02	56.25	14.67	0.871
*AsSWEET12-1*	804	68,695.44	5.03	58.07	17.91	0.799
*AsSWEET12-2*	687	57,957.26	5.08	52.75	17.61	0.860
*AsSWEET13-1*	675	55,438.35	5.08	51.67	20.59	1.004
*AsSWEET13-2*	831	69,651.03	5.03	52.59	16.49	0.814
*AsSWEET13-3*	657	55,396.52	5.08	52.79	16.89	0.892
*AsSWEET14-1*	858	71,819.04	5.01	55.83	18.65	0.853
*AsSWEET14-2*	1128	92,667.56	4.99	48.41	19.95	0.909
*AsSWEET15-1*	390	33,558.76	5.16	53.69	13.59	0.927
*AsSWEET15-2*	879	72,421.47	5.03	47.57	17.41	0.731
*AsSWEET16*	936	76,825.64	5.02	53.29	17.74	0.663
*AsSWEET16-1*	414	34,697.08	5.21	50.47	20.53	0.923
*AsSWEET16-2*	844	70,557.10	4.78	50.61	18.79	0.696
*AsSWEET17-1*	750	62,989.42	5.08	52.43	20.27	0.714
*AsSWEET17-2*	753	62,918.27	5.08	51.34	20.85	0.728

Note: Gene names in the table are sorted in ascending order.

**Table 2:** Secondary structure content of the ammonium transporter AsSWEET protein family.

Gene Name	Alpha Helix (Hh)	Beta Turn (Tt)	Random Coil (Ee)	Extended Strand (Cc)
*AsSWEET1a*	48.66	3.83	31.03	16.47
*AsSWEET1a-1*	55.26	3.01	26.69	16.36
*AsSWEET1b*	47.33	4.12	29.22	19.05
*AsSWEET2a-1*	36.54	8.65	35.10	25.81
*AsSWEET2a-2*	41.61	10.22	20.44	21.83
*AsSWEET2b-1*	45.78	8.67	26.00	19.34
*AsSWEET2b-2*	36.59	6.71	34.45	27.74
*AsSWEET3b*	41.59	3.27	38.79	23.98
*AsSWEET4*	42.74	3.23	30.65	22.26
*AsSWEET4-1*	43.55	4.03	26.61	23.18
*AsSWEET4-2*	51.18	7.87	21.26	23.39
*AsSWEET6a-1*	39.19	4.05	34.68	18.48
*AsSWEET6a-2*	39.84	4.47	31.71	16.48
*AsSWEET6a-3*	43.23	4.37	30.57	17.83
*AsSWEET11*	40.74	3.70	33.64	19.56
*AsSWEET11-1*	37.75	4.64	34.44	19.19
*AsSWEET11-2*	48.84	1.55	31.78	19.72
*AsSWEET12-1*	37.45	0.75	43.82	17.98
*AsSWEET12-2*	55.26	2.63	26.75	19.71
*AsSWEET13-1*	41.07	5.36	35.27	37.98
*AsSWEET13-2*	47.10	3.26	31.16	18.30
*AsSWEET13-3*	40.37	1.83	38.07	20.02
*AsSWEET14-1*	45.96	3.86	34.39	19.69
*AsSWEET14-2*	40.96	5.17	34.69	20.40
*AsSWEET15-1*	26.23	7.07	46.68	22.07
*AsSWEET15-2*	42.47	3.42	37.67	16.44
*AsSWEET16*	44.63	3.68	33.44	15.04
*AsSWEET16-1*	30.23	2.33	29.46	21.91
*AsSWEET16-2*	42.49	3.66	34.80	15.35
*AsSWEET17-1*	49.80	4.02	29.72	15.79
*AsSWEET17-2*	38.40	3.60	37.60	16.40

Note: Gene names in the table are sorted in ascending order.

**Table 3:** Prediction of subcellular localization results of *AsSWEET*.

Gene Name	Subcellular Localization
*AsSWEET1a*	Plasma membrane
*AsSWEET1a-1*	Plasma membrane
*AsSWEET1b*	Tonoplast
*AsSWEET2a-1*	Plasma membrane
*AsSWEET2a-2*	Tonoplast
*AsSWEET2b-1*	Plasma membrane
*AsSWEET2b-2*	Nucleus
*AsSWEET3b*	Chloroplast
*AsSWEET4*	Extracellular matrix
*AsSWEET4-1*	Extracellular matrix
*AsSWEET4-2*	Tonoplast
*AsSWEET6a-1*	Plasma membrane
*AsSWEET6a-2*	Plasma membrane
*AsSWEET6a-3*	Plasma membrane
*AsSWEET11*	Plasma membrane
*AsSWEET11-1*	Plasma membrane
*AsSWEET11-2*	Plasma membrane
*AsSWEET12-1*	Plasma membrane
*AsSWEET12-2*	Chloroplast
*AsSWEET13-1*	Plasma membrane
*AsSWEET13-2*	Plasma membrane
*AsSWEET13-3*	Plasma membrane
*AsSWEET14-1*	Plasma membrane
*AsSWEET14-2*	Plasma membrane
*AsSWEET15-1*	Tonoplast
*AsSWEET15-2*	Plasma membrane
*AsSWEET16*	Plasma membrane
*AsSWEET16-1*	Tonoplast
*AsSWEET16-2*	Tonoplast
*AsSWEET17-1*	Tonoplast
*AsSWEET17-2*	Tonoplast

Note: Gene names in the table are sorted in ascending order.

## Data Availability

Data is contained within the article and Supplementary Materials.

## Supplementary Materials

The following supporting information can be downloaded at https://www.mdpi.com/article/10.3390/ijms26136438/s1.
