# Supplementary material for: Identification and Expression Pattern Analysis of AsSWEET Gene Family in Achnatherum splendens
Source: Int J Mol Sci. 2025 Jul 4;26(13):6438. doi: 10.3390/ijms26136438 (PMC12249787; doi:10.3390/ijms26136438)
Supplement: Supplementary file 1 [file ijms-26-06438-s001.zip › ijms-3673918-supplementary.pdf]

Supplementary Materials

Table S1 qRT-PCR primers

Gene name	Forward primer (5′–3′)	Reverse primer (5′–3′)
AsSWEET1a	TCTTCTTCGGGGTTTCAGGC	TCACCAGGATGTTGTTCGGG
AsSWEET1a-1	GCCGGTAGTCACCTTTTGGA	TCACCAGGATGTTGTTCGGG
AsSWEET1b	CAGAGGAATTCTCCGGGGTG	TCGACGCGAAGATGAGGAAG
AsSWEET2a-1	GAAGAGGCTGAAGGTCTCCG	ATGACACCCAAGACAGTGCC
AsSWEET2a-2	GTTTGATCACGAAGCTCGGC	GGCATGTACTCCACGCTCTT
AsSWEET2b-1	TCATGGACTCCCTCTCCCTG	GGTCGATTTGGCCTTCAGGA
AsSWEET2b-2	GTCATCCGTTTTCCGGAGGT	GGTCGATTTGGCCTTCAGGA
AsSWEET3b	ACAGGAAAAGCGATGGAGCA	CTGGCTTATTTCCAGCGAGC
AsSWEET4	CCAGACAAAGAGCGTGGAGT	AAAGGTCGAAGCGGATGAGG
AsSWEET4-1	AAGAGCGTGGAGTACATGCC	TCTGCTGCGTCGACTTGTAG
AsSWEET4-2	TAGGCAATGGAACTGCCCTG	AGAGCACCCACATCATGCAG
AsSWEET6a-1	CGACCTCTACGTCACGATCC	CGACCTCTACGTCACGATCC
AsSWEET6a-2	TCGTCACCATCAACGGAGTC	ACGATCATGGAGCGCTTCTC
AsSWEET6a-3	ATGGAATCGGGCTCGTCATC	GCCGAATATGACGCAGAGGA
AsSWEET11	CAAGACCAAGAGCGTGGAGT	GGTACCAGAAGTAGAGCGCC
AsSWEET11-1	CGCGTGGTTCTTCTACGGAT	CTCAGTCACGGAATCGTCGG
AsSWEET11-2	CTTCTTCCTCACCCTGAGCG	AGTAGAGCGTCATCTGTGCG
AsSWEET12-1	ACGAGGACGAGGTACAGACA	TGCGGTGAGAGGAGGAAATG
AsSWEET12-2	GTCAACGGAGTCGTTCCAGT	CAACGAGGTAGACGGACTCG
AsSWEET13-1	CGAGGCAACGTCATCTCCTT	GTCTTGACCAGCGCGTAGTA
AsSWEET13-2	ACATCGTCATGTACCTCGCC	CATGAAGACGCTGACGGAGA
AsSWEET13-3	TGGTCAAGTCCAACGAGAGC	CATGAAGACGCTGACGGAGA
AsSWEET14-1	ATCGTCGTCTACCTCGCCTA	AGAAGGGCATGAACTCCACG
AsSWEET14-2	GTTCCAGTCGGTTCCCTACG	AGGATCTTGGCCGTGAACAG
AsSWEET15-1	GCTGTGGATGTACTACGCCT	AACAGCTTTGCCGTGAGGAG
AsSWEET15-2	CTCCTCACGGCGAAACTCTT	GTCCGGATCACAAGCCTGAT
AsSWEET16	GTGAGGAACAAGAGCACCGA	GGGGCATACGCGAGATAGAG
AsSWEET16-1	TGGTGTTTTCGGAGTCGTGT	TGTACTCCACGCTCCTTGTG
AsSWEET16-2	GCCTACTCCATGCTCGTCAA	CTTGTTCCTGTACGCCAGGT
AsSWEET17-1	CCGTCATGGAGGCCATCTAC	TGTGGCTGAGAACACGACTC
AsSWEET17-2	TTGGGCTGTCTACGCCATAC	AGAAGGGAAGCCGAAACCTG

* Note: Gene names in the table are sorted in ascending order.

Table S2. Link agereaction system

Group component	Add
PCR recovery product	4 μL
Recombinase	5 μL
Linearized vector	1 μL
Total voume	10 μL

Mix well and perform ligation for 30 min

Table S3. Colony PCR system

Group component	Add
2×Taq PCR Mix	5 μL
Primer-F(10 mM)	0.5 μL
Primer-R(10 mM)	0.5 μL
ddH_20	3 μL
Total voume	10 μL

Table S4. PCR reaction procedure

Step	reaction conditions
Step1	95 ℃，3 min；
Step2	95 ℃，30 s；
Step3	58 ℃，30 s；
Step4	72 ℃，1 min，Repeat Step 2 for 35 cycles
Step6	72 ℃，10 min；
Step7	4 ℃，1 h

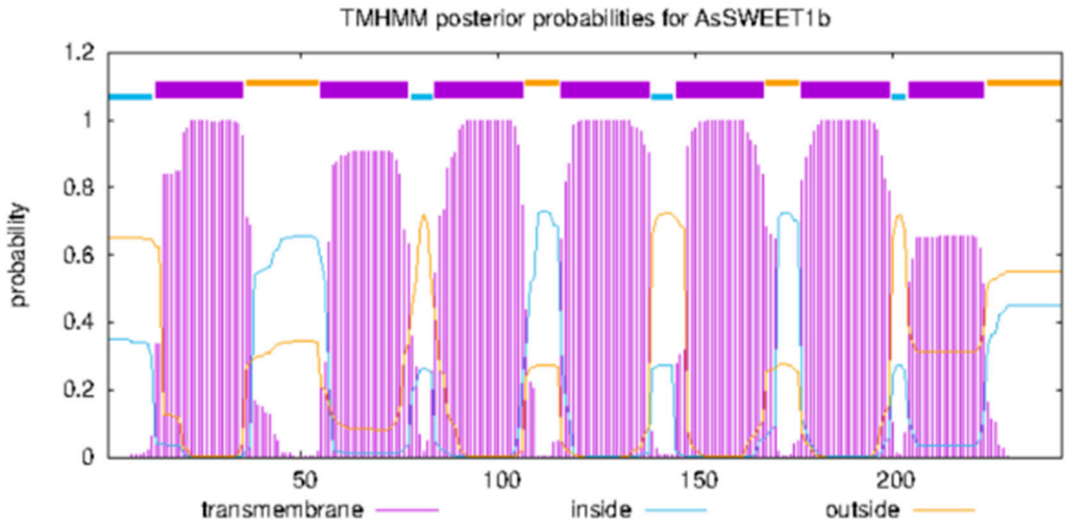
TMHMM posterior probabilities for AsSWEET1b
probability
1.2
1
0.8
0.6
0.4
0.2
0
50
100
150
200
transmembrane
inside
outside

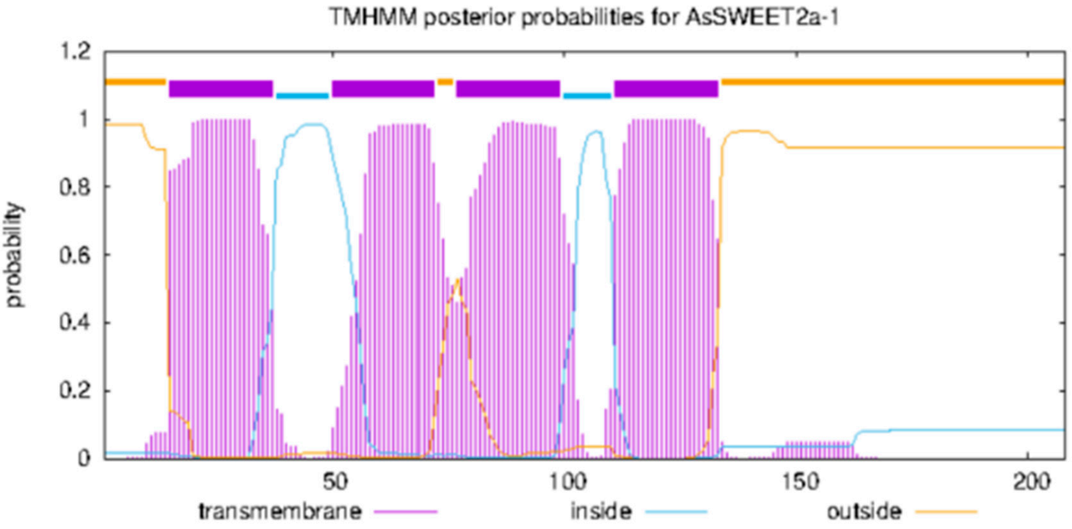
TMHMM posterior probabilities for AsSWEET2a-1
probability
1.2
1
0.8
0.6
0.4
0.2
0
50
100
150
200
transmembrane
inside
outside

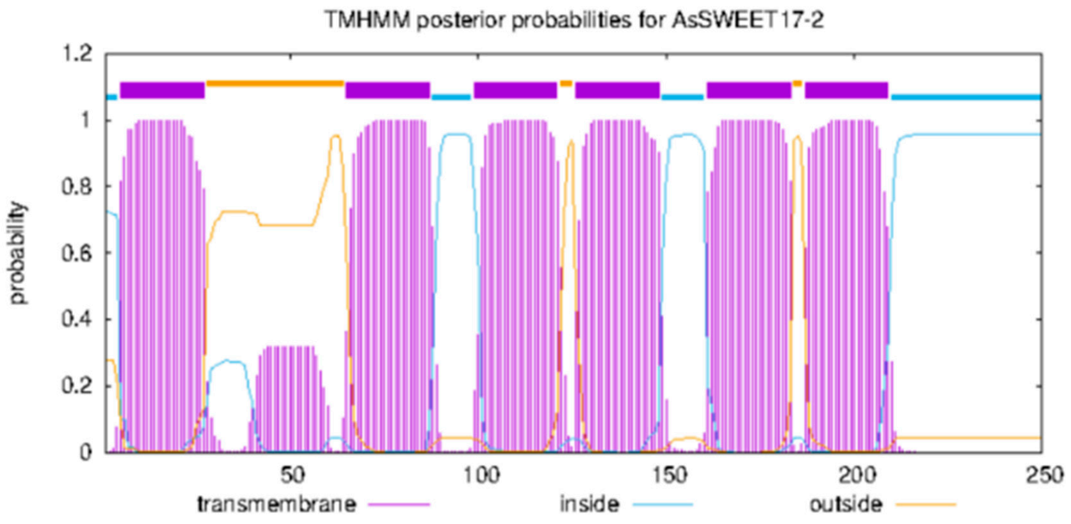
TMHMM posterior probabilities for AsSWEET17-2
probability
1.2
1
0.8
0.6
0.4
0.2
0
50
100
150
200
250
transmembrane
inside
outside

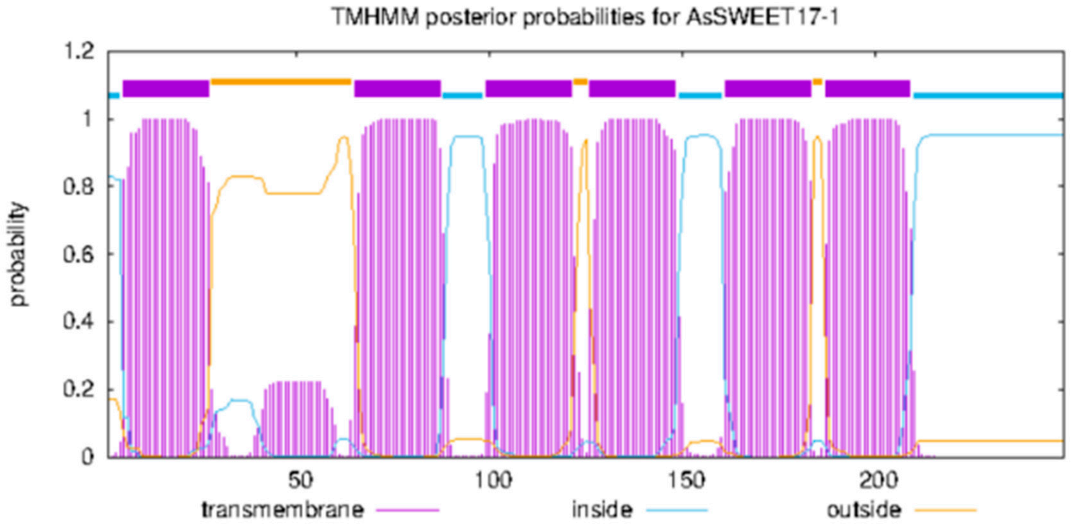
TMHMM posterior probabilities for AsSWEET17-1
probability
1.2
1
0.8
0.6
0.4
0.2
0
50
100
150
200
transmembrane
inside
outside

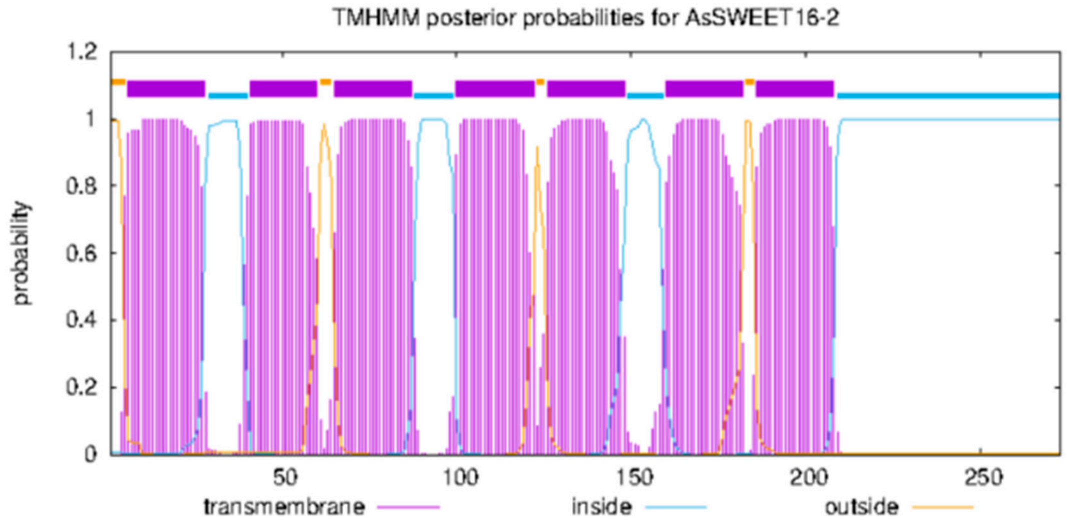
TMHMM posterior probabilities for AsSWEET16-2
probability
1.2
1
0.8
0.6
0.4
0.2
0
50
100
150
200
250
transmembrane
inside
outside

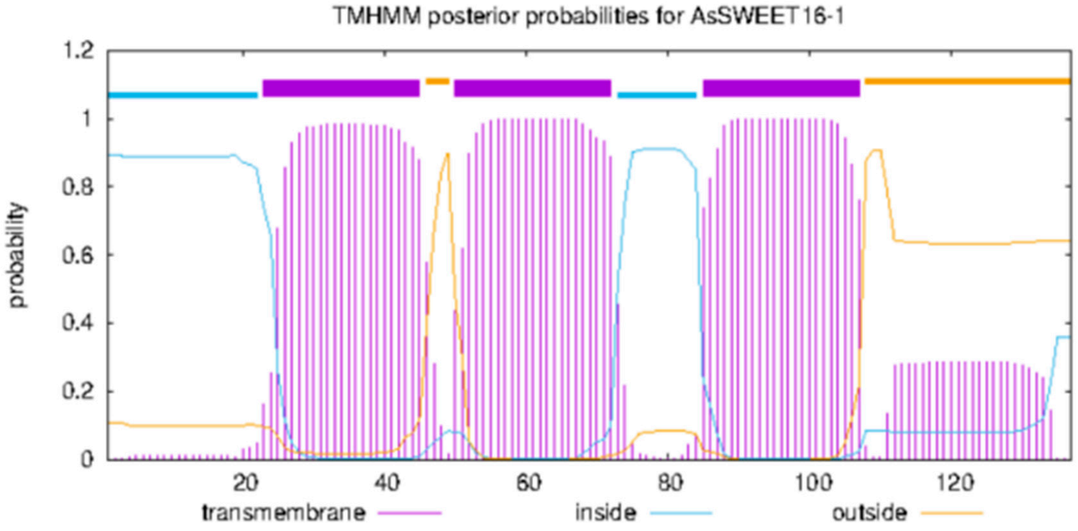
TMHMM posterior probabilities for AsSWEET16-1
probability
transmembrane
inside
outside

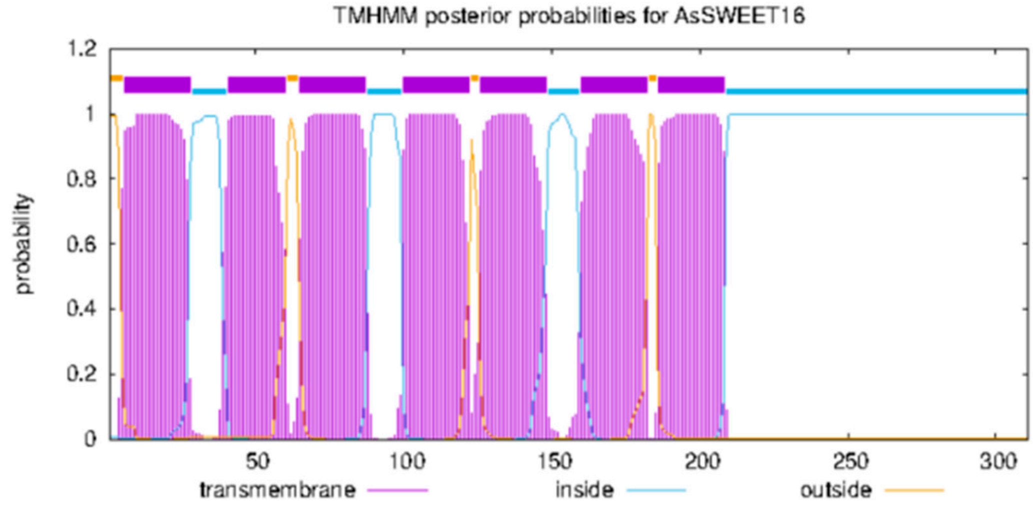
TMHMM posterior probabilities for AsSWEET16
probability
transmembrane
inside
outside

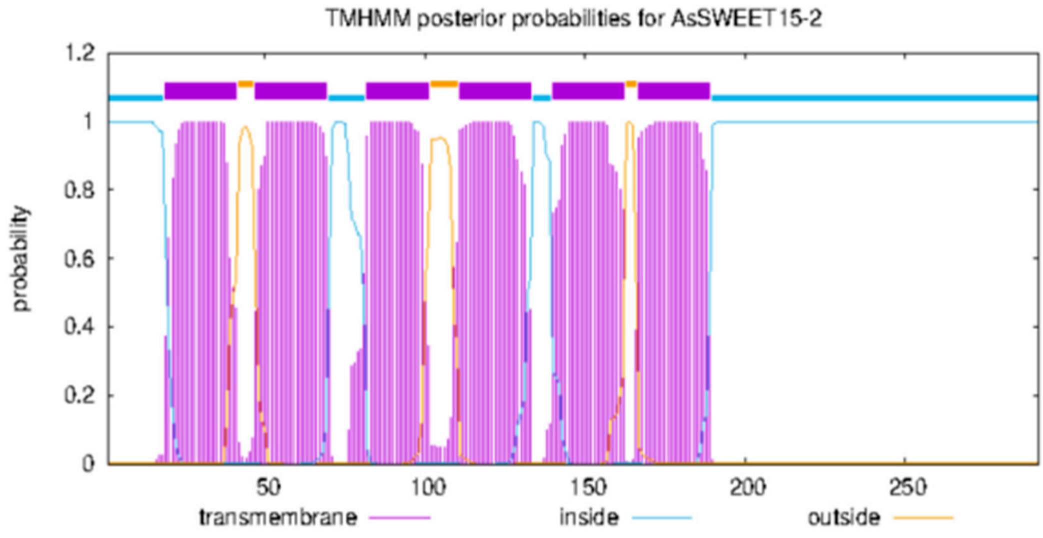
TMHMM posterior probabilities for AsSWEET15-2
probability
transmembrane
inside
outside

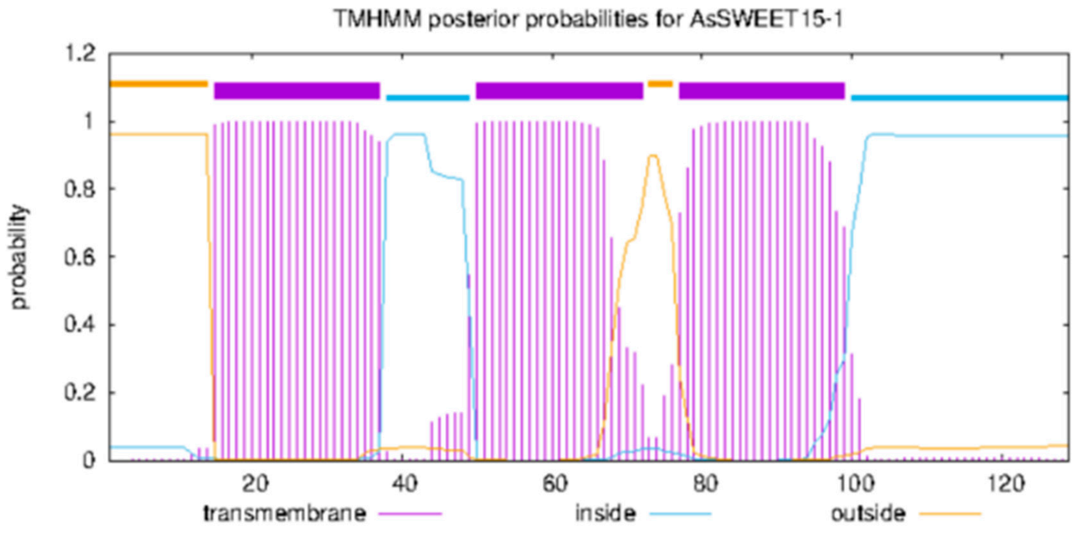
TMHMM posterior probabilities for AsSWEET15-1
probability
1.2
1
0.8
0.6
0.4
0.2
0
20
40
60
80
100
120
transmembrane
inside
outside

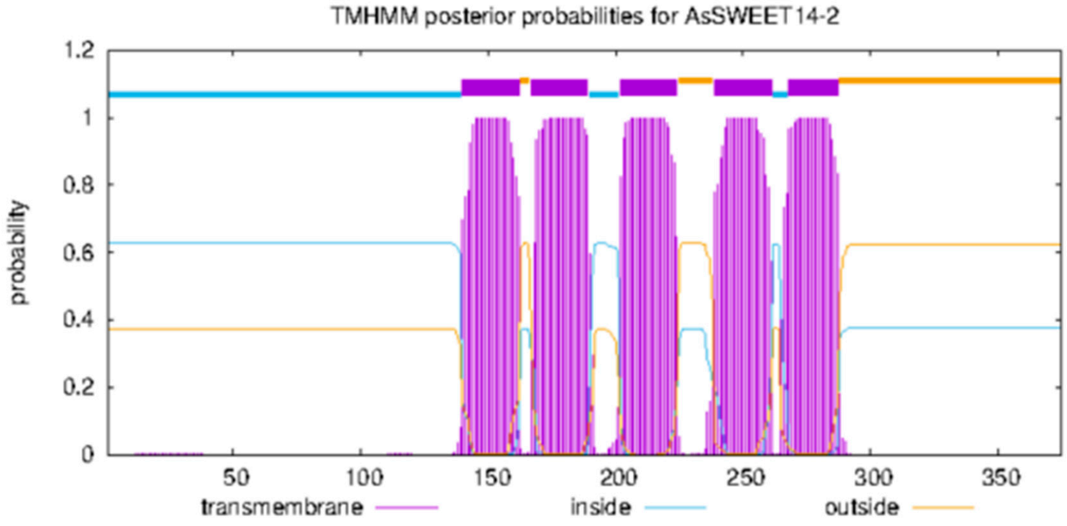
TMHMM posterior probabilities for AsSWEET14-2
probability
1.2
1
0.8
0.6
0.4
0.2
0
50
100
150
200
250
300
350
transmembrane
inside
outside

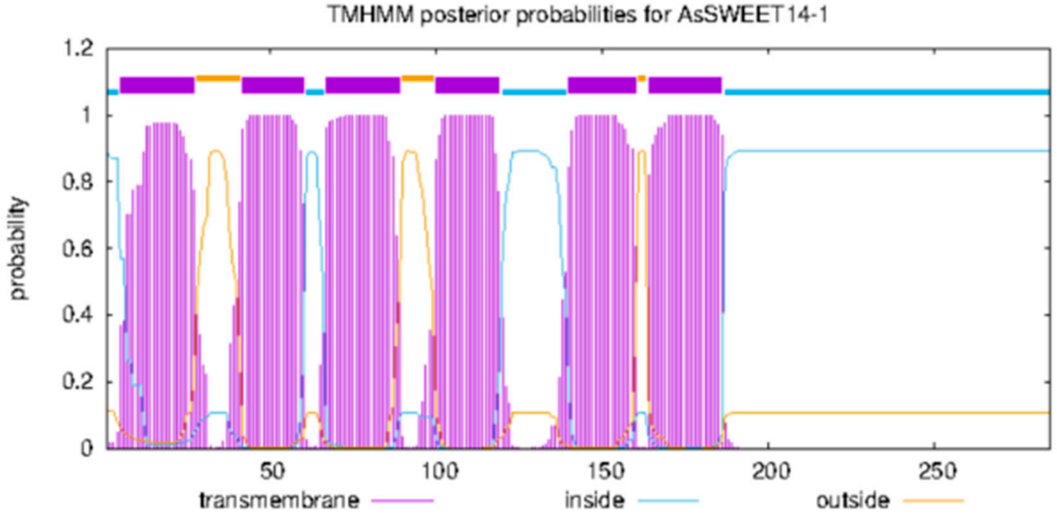
TMHMM posterior probabilities for AsSWEET14-1
probability
1.2
1
0.8
0.6
0.4
0.2
0
50
100
150
200
250
transmembrane
inside
outside

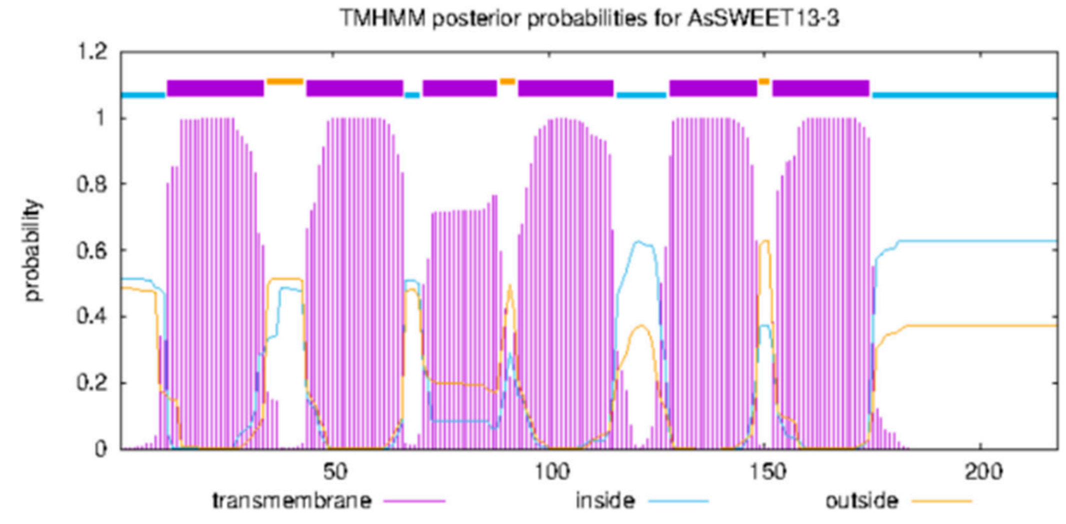
TMHMM posterior probabilities for AsSWEET13-3
probability
transmembrane
inside
outside

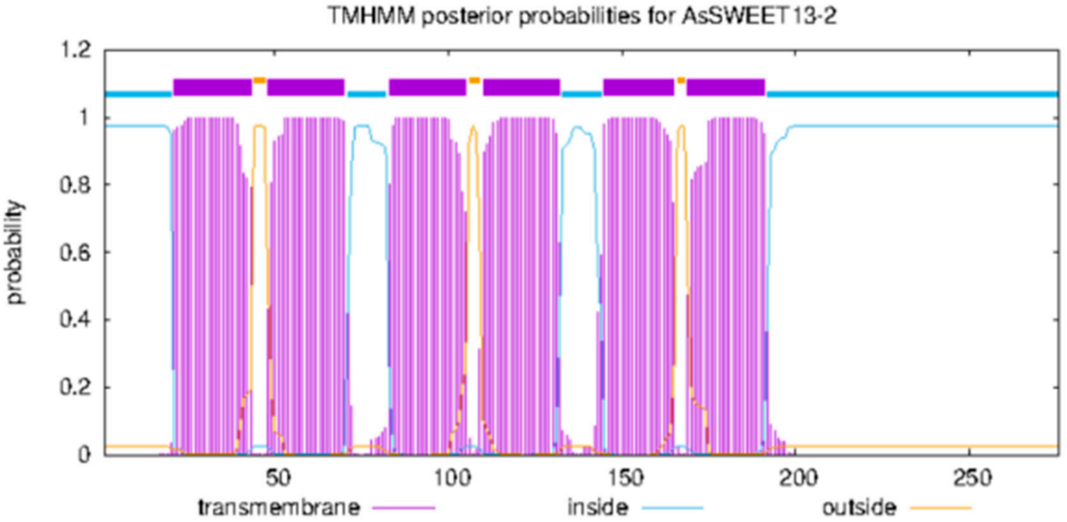
TMHMM posterior probabilities for AsSWEET13-2
probability
transmembrane
inside
outside

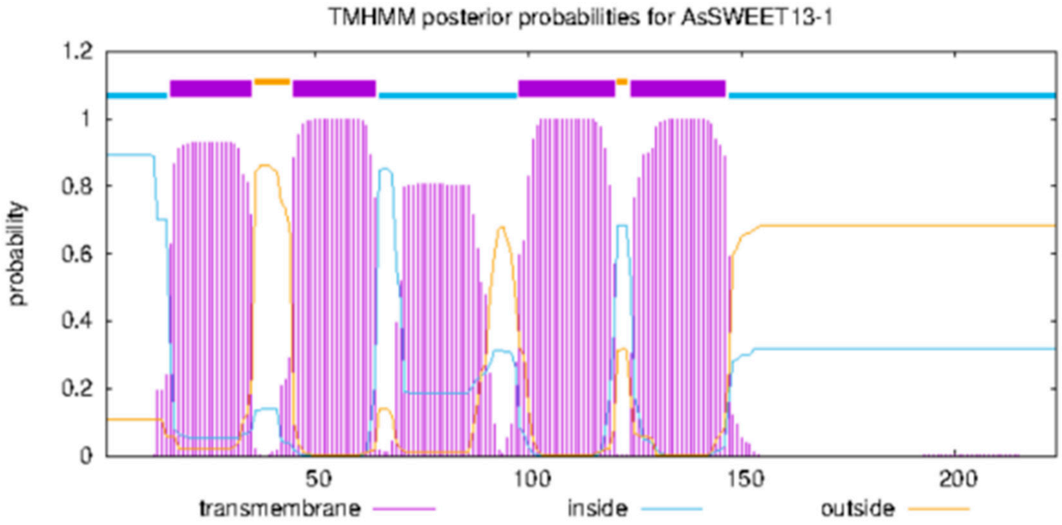
TMHMM posterior probabilities for AsSWEET13-1
probability
transmembrane
inside
outside

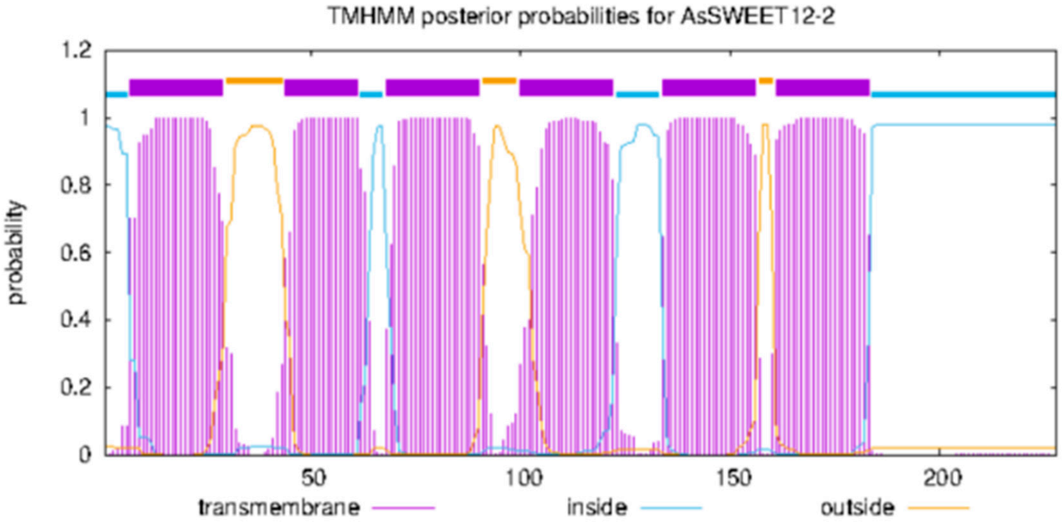
TMHMM posterior probabilities for AsSWEET12-2
probability
transmembrane
inside
outside

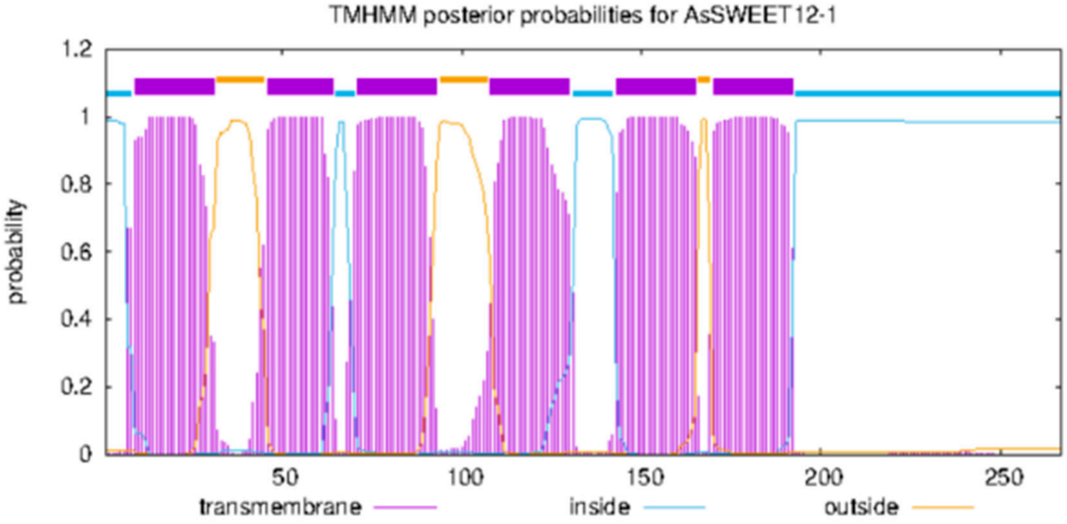
TMHMM posterior probabilities for AsSWEET12-1
probability
transmembrane
inside
outside

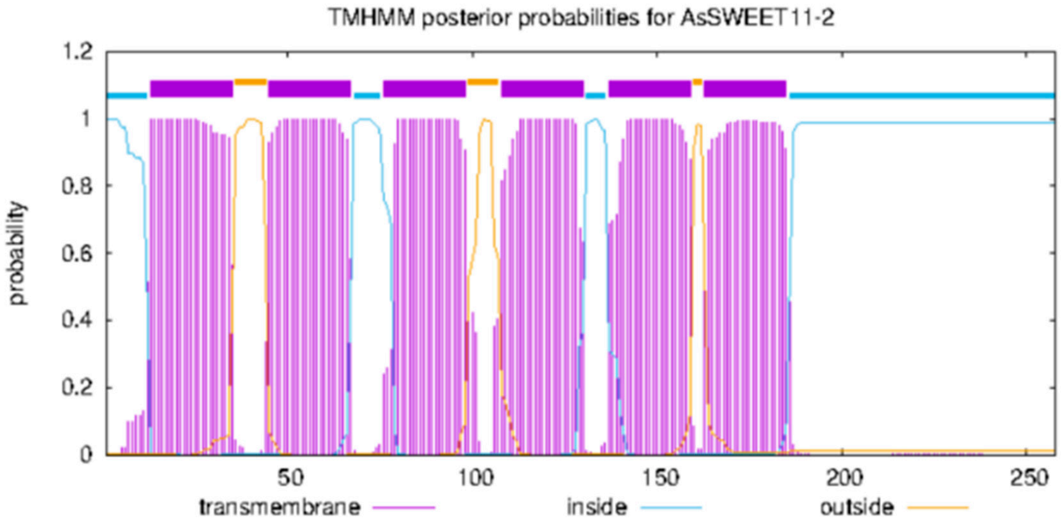
TMHMM posterior probabilities for AsSWEET11-2
probability
transmembrane
inside
outside

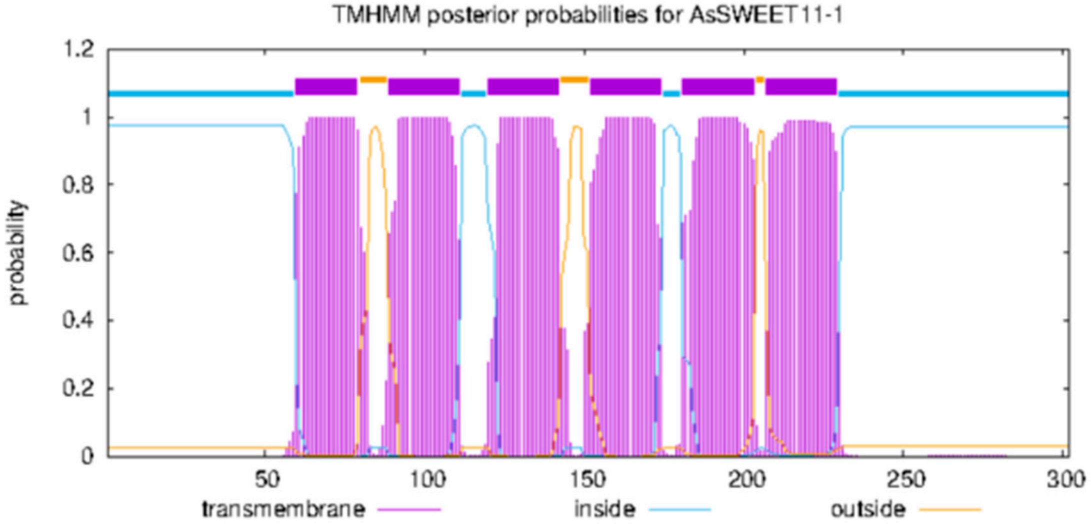
TMHMM posterior probabilities for AsSWEET11-1
probability
transmembrane
inside
outside

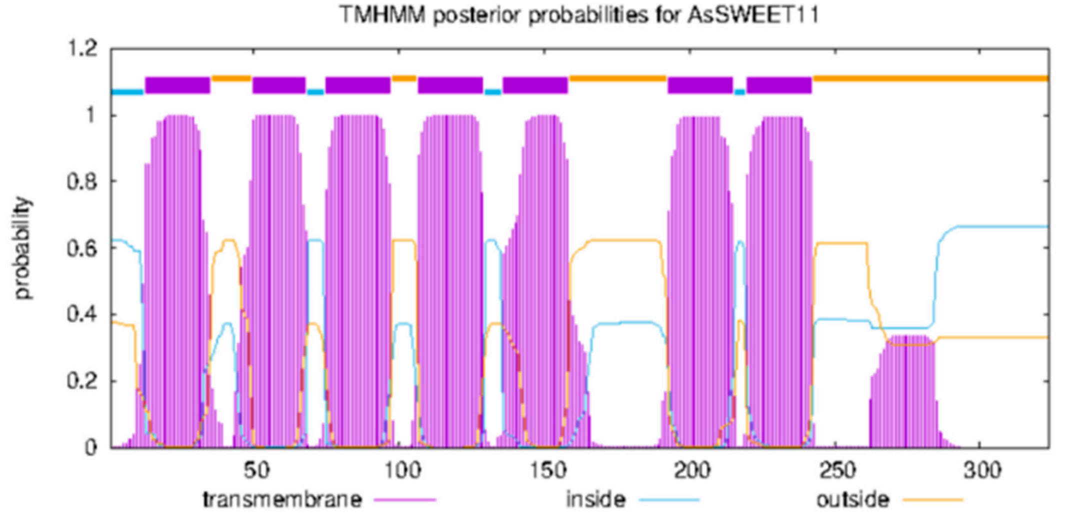
TMHMM posterior probabilities for AsSWEET11
probability
transmembrane
inside
outside

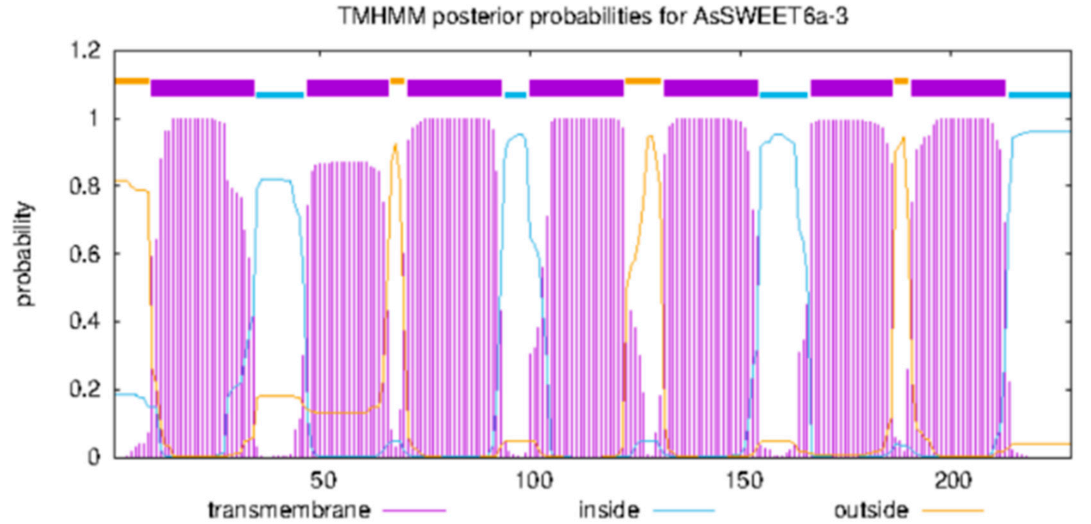
TMHMM posterior probabilities for AsSWEET6a-3
probability
transmembrane
inside
outside

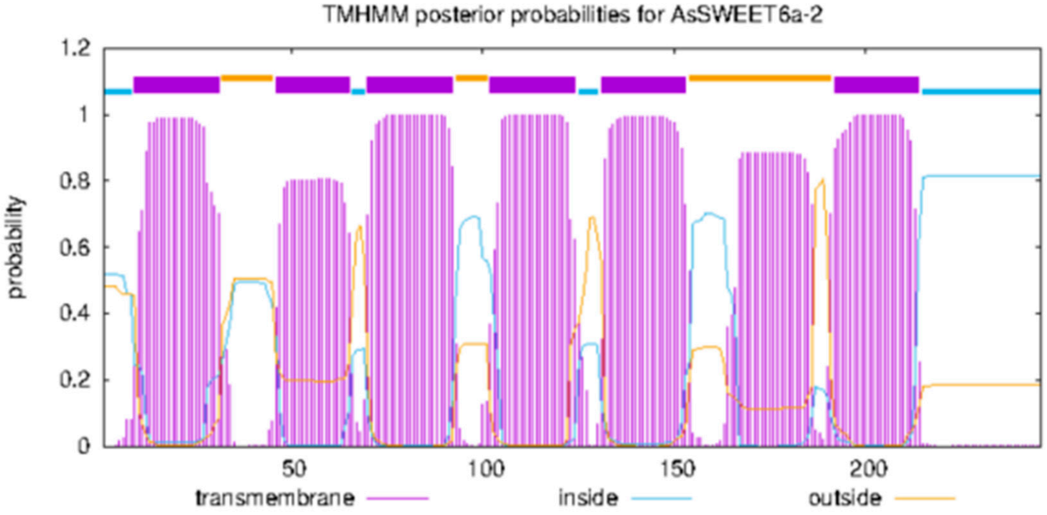
TMHMM posterior probabilities for AsSWEET6a-2
1.2
1
0.8
0.6
0.4
0.2
0
probability
50
100
150
200
transmembrane
inside
outside

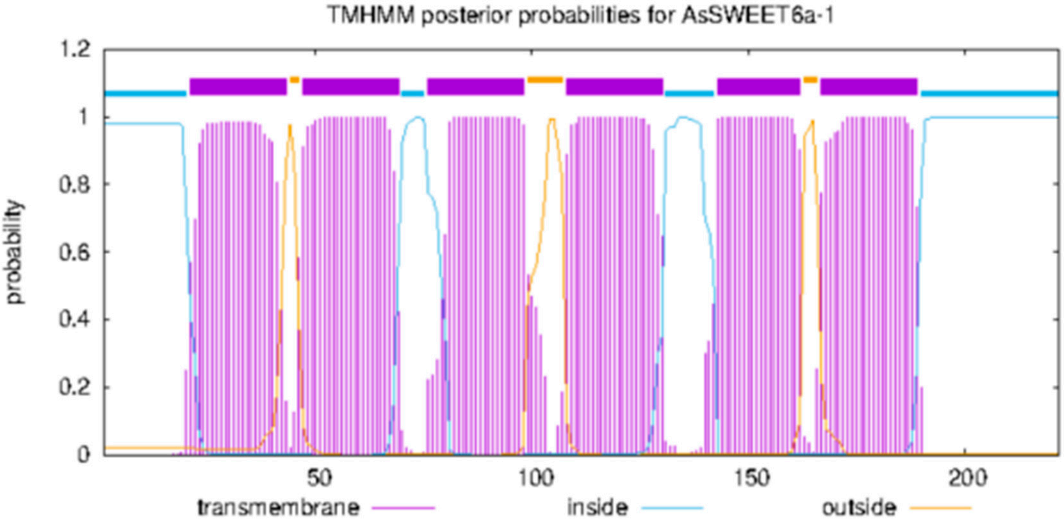
TMHMM posterior probabilities for AsSWEET6a-1
1.2
1
0.8
0.6
0.4
0.2
0
probability
50
100
150
200
transmembrane
inside
outside

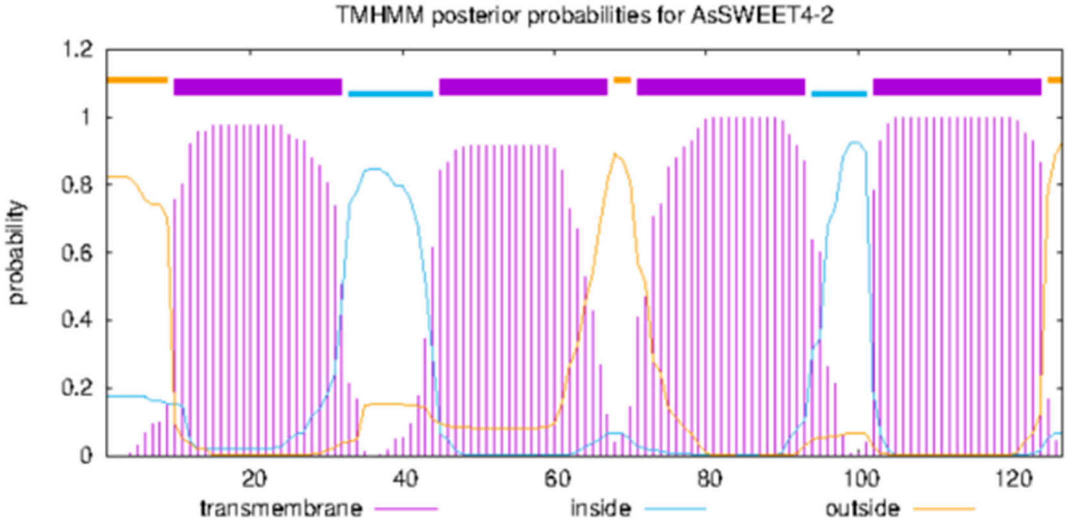
TMHMM posterior probabilities for AsSWEET4-2
1.2
1
0.8
0.6
0.4
0.2
0
probability
20
40
60
80
100
120
transmembrane
inside
outside

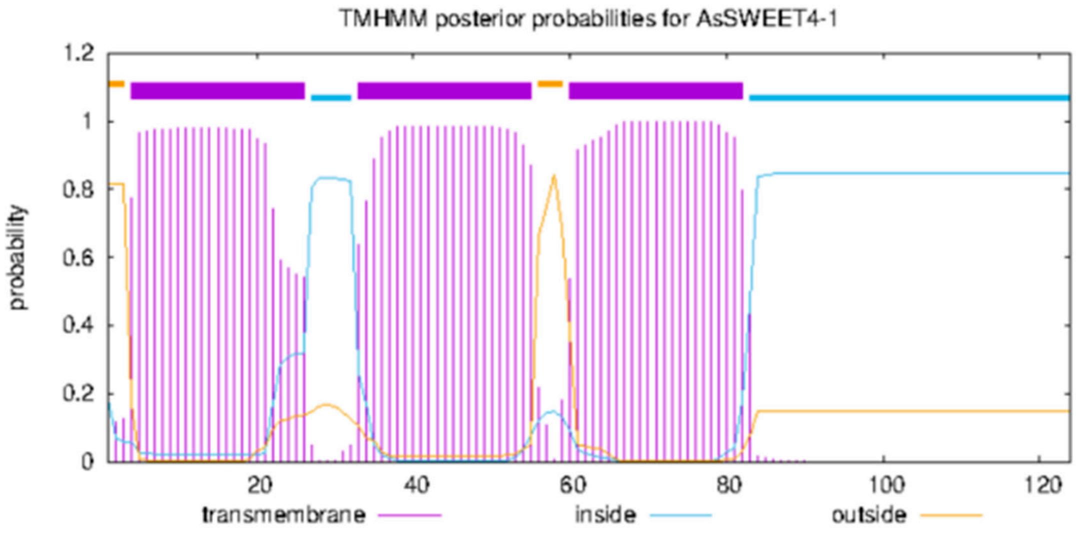
TMHMM posterior probabilities for AsSWEET4-1
probability
transmembrane
inside
outside

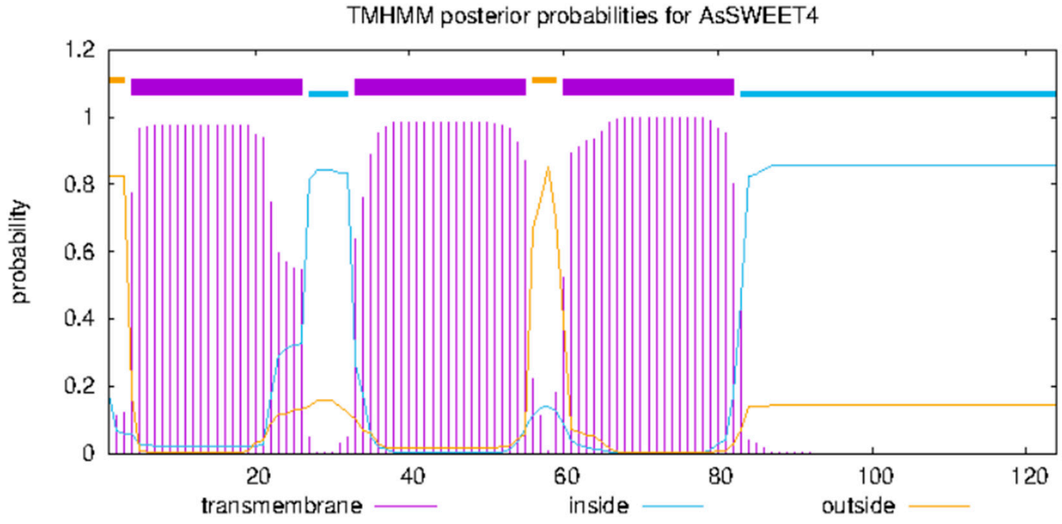
TMHMM posterior probabilities for AsSWEET4
probability
transmembrane
inside
outside

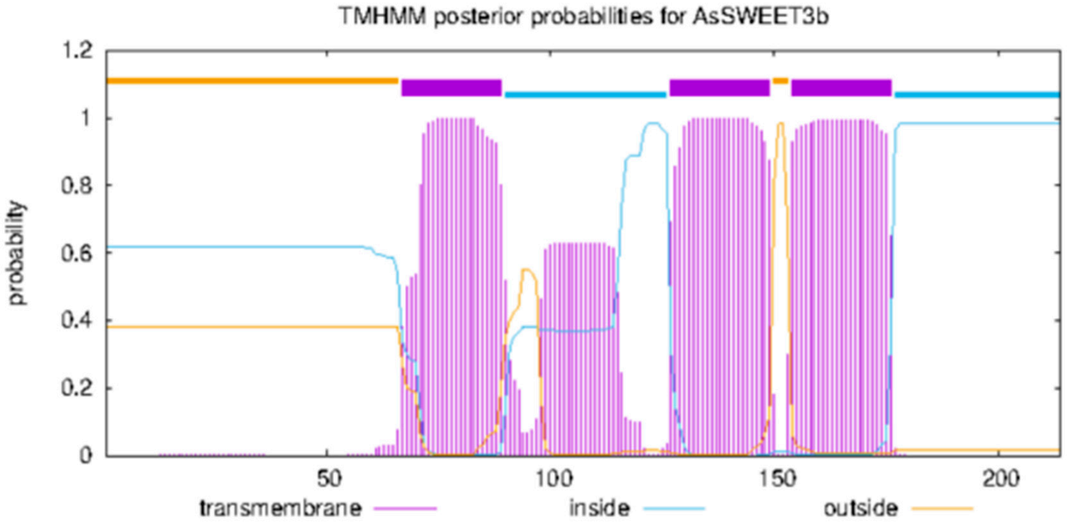
TMHMM posterior probabilities for AsSWEET3b
probability
transmembrane
inside
outside

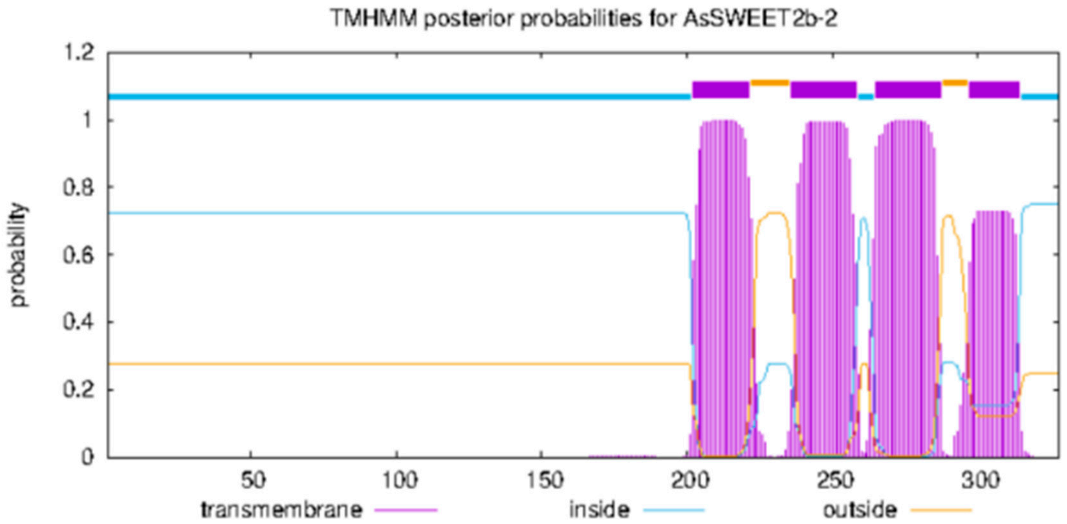
TMHMM posterior probabilities for AsSWEET2b-2
probability
transmembrane
inside
outside

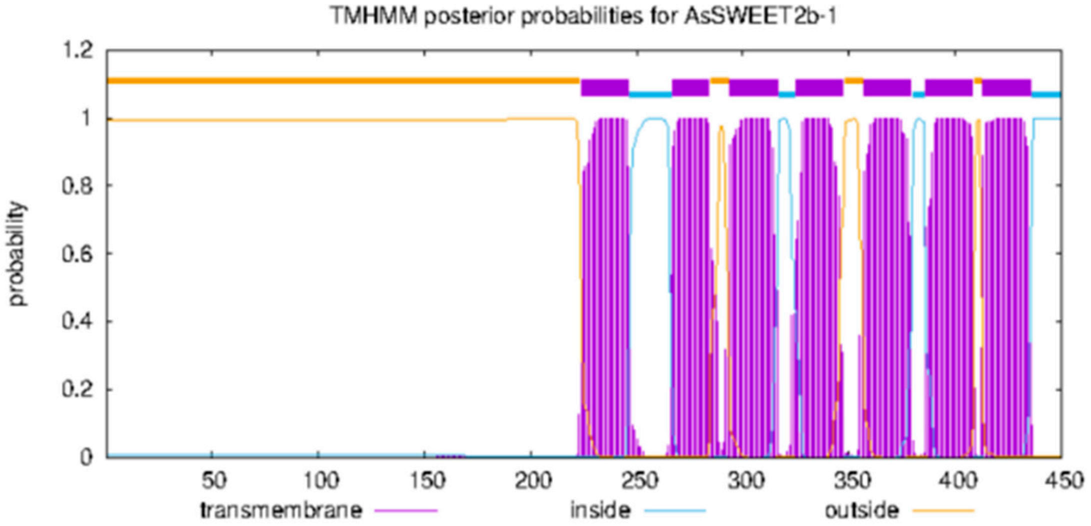
TMHMM posterior probabilities for AsSWEET2b-1
probability
transmembrane
inside
outside

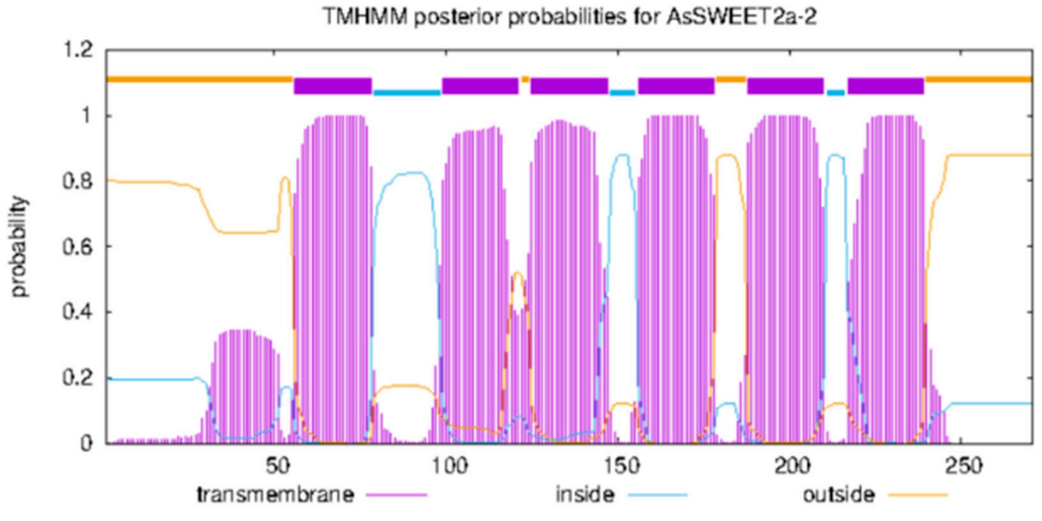
TMHMM posterior probabilities for AsSWEET2a-2
probability
transmembrane
inside
outside

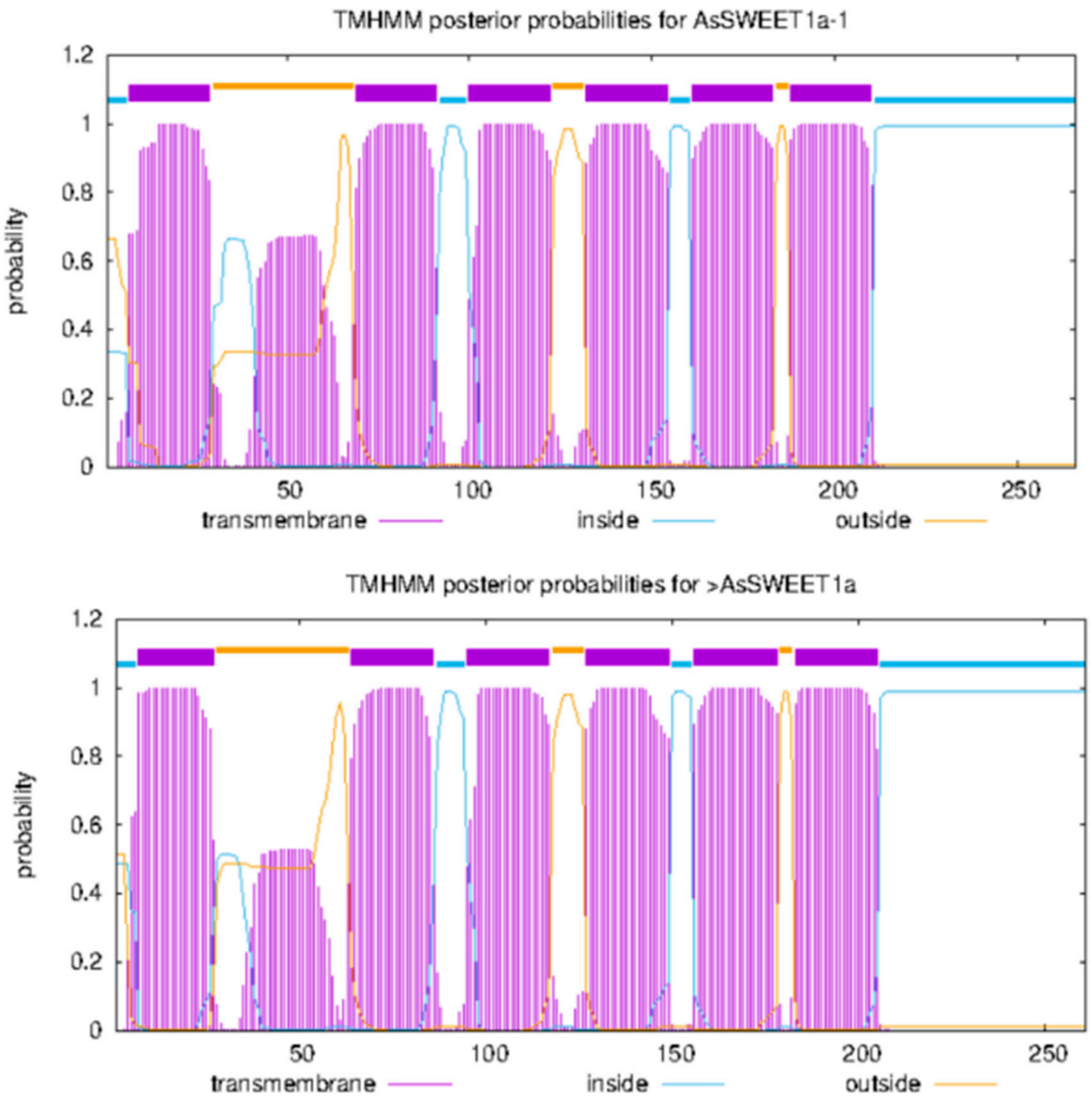

Figure S1. transmembrane structural domain
